# Real-time 3D cardiac tissue implementation for arrhythmia detection and management

**DOI:** 10.3389/fbioe.2026.1840143

**Published:** 2026-07-03

**Authors:** Anwu Huang, Wei Shen, Lianglei Hou, Yunlong Wang, Xiaotian Pan, Bin Lin

**Affiliations:** 1 Department of Cardiology, Wenzhou Central Hospital, Wenzhou, Zhejiang, China; 2 Institute of Intelligent Media Computing, Hangzhou Dianzi University, Hangzhou, China

**Keywords:** Aliev-Panfilov model, arrhythmia management, cardiac tissue simulation, digital twin-inspired frame work, fixed-point arithmetic, FPGA, real-time systems

## Abstract

**Introduction:**

This paper presents a high-performance, real-time hardware implementation of 3D cardiac tissue for the detection and management of complex arrhythmias.

**Methods:**

Utilizing the Aliev-Panfilov reaction-diffusion model, we simulate electrical wave propagation in a 200×200×3 lattice comprising 120,000 nodes. To achieve real-time performance on a Xilinx Virtex-7 Field-Programmable Gate Array (FPGA), we developed a massively parallel architecture based on a multi-precision fixed-point arithmetic framework. This approach ensures high physiological fidelity with a Mean Squared Error (MSE) of 10 compared to 64-bit floating-point simulations, while significantly optimizing resource utilization.

**Results:**

Operating at 125 MHz, the proposed processor achieves a full lattice update in 0.96 ms, providing a 56-fold acceleration margin over biological real-time dynamics. Furthermore, the system incorporates a hardware-level supervisory layer that monitors wave-break density and dominant frequency to classify cardiac states into Periodic Spiral (PS), Quasi-Periodic (QS), and Spiral Turbulence (ST).

**Discussion:**

This “digital twin-inspired framework” framework enables sub-millisecond, closed-loop therapeutic interventions, facilitating the autonomous management of life-threatening arrhythmic conditions with a total power consumption of 5.42 W.

## 1 Introduction

Cardiac arrhythmias, characterized by abnormal electrical activity in the heart, remain one of the leading causes of morbidity and mortality worldwide (Ha et al., 2025; Busia et al., 2024). These pathological conditions, ranging from atrial fibrillation to life-threatening ventricular fibrillation, arise from the formation of chaotic electrical re-entrant waves, often termed spiral waves (Bashar et al., 2020). The transition from stable sinus rhythm to complex spatiotemporal turbulence can lead to sudden cardiac arrest within minutes. Understanding the underlying mechanisms of these wave dynamics is crucial for developing effective therapeutic strategies and improving patient survival rates (Chu et al., 2026; Lampert and Harmon, 2026; Lertwanakarn, 2026).

To study these complex phenomena, mathematical modeling of cardiac electrophysiology has become an indispensable tool. When extended to three-dimensional (3D) lattices, these models can accurately replicate the transmural propagation of electrical signals (Gomes et al., 2014; Sun M. et al., 2025; Grandits et al., 2023; Martinez et al., 2026). However, simulating a 3D cardiac tissue with high resolution involves solving thousands of coupled differential equations, which imposes an extreme computational burden on traditional general-purpose processors (Kogan et al., 2026).

In (Viola et al., 2023), a comprehensive cardiovascular digital twin-inspired frame work was developed using high-performance Graphics processing unit (GPU) acceleration to replicate multi-physics heart dynamics. This model integrates electrophysiology, mechanics, and hemodynamics, enabling *in silico* clinical trials to study synthetic patient cohorts and evaluate the efficacy of prosthetic devices like pacemakers. In (Hariri et al., 2025), a systematic analysis of 59 studies identifies hybrid Field-Programmable Gate Array (FPGA)-Application-specific integrated circuit (ASIC) and Edge Artificial Intelligence (AI) architectures as the optimal hardware solutions for real-time cardiovascular monitoring. This work benchmarks energy efficiency and clinical accuracy to guide the development of next-generation diagnostic systems. In (Karataş et al., 2022), eight arrhythmic ElectroCardioGraphy (ECG) signals were modeled and implemented on a Zynq-7000 FPGA for biomedical calibration. The system achieves a high operating frequency of 651.827 MHz with low Mean Squared Error (MSE) compared to the MIT-BIH database. In (Pan et al., 2025), a neural network-based classification algorithm was implemented on an FPGA using Verilog Hardware Description Language (HDL) to detect Sudden Cardiac Arrest (SCA) from Heart Rate Variability (HRV) metrics. The proposed hardware-accelerated model achieves an accuracy of 96.23% and was successfully verified through Register-Transfer Level (RTL) simulation in Vivado. In (Ghanbarpour et al., 2025), a novel method for reducing nonlinear terms in cardiac Purkinje fiber cell models was implemented on a Virtex-7 FPGA, achieving a 3.49-fold frequency improvement. The design demonstrates high scalability for real-time simulations, successfully modeling networks of up to 4500 cells with superior hardware efficiency.

The need for real-time monitoring and instantaneous intervention necessitates the transition from software-based simulations to dedicated hardware implementations (Haghiri et al., 2026a; Haghiri et al., 2026b; Majidifar et al., 2024). Conventional CPUs and GPUs often suffer from non-deterministic latencies and high power consumption, making them unsuitable for closed-loop medical devices. FPGAs offer a promising alternative by providing massive parallelism, low-latency processing, and high energy efficiency (Majidifar et al., 2022; Majidifar et al., 2023; Ghanbarpour et al., 2023a; Chaudhary et al., 2024; Ghanbarpour et al., 2023b; Sun J. et al., 2025; Chen et al., 2024). Implementing a “digital twin-inspired frame work” of cardiac tissue on an FPGA allows for the deterministic, real-time tracking of wave dynamics, which is essential for triggering timely defibrillation or inhibitory stimuli (Crane et al., 2025).

The primary contributions of this research, which distinguishes it from existing cardiac modeling works, are as follows:

First, we propose a massively parallel hardware architecture for the 3D Aliev-Panfilov model, enabling high-fidelity simulation of a 200×200×3 lattice. This design achieves a 56-fold acceleration margin over biological real-time dynamics, providing a critical temporal window for predictive analysis and instantaneous therapeutic decision-making. Second, to address the trade-off between hardware resource constraints and numerical stability, a custom multi-precision fixed-point arithmetic framework is developed. This framework maintains a high accuracy with an MSE of $10^{-2}$ relative to double-precision software models, while significantly optimizing the consumption of Digital Signal Processing (DSP) slices and LookUp Tables (LUTs) on the Virtex-7 FPGA. Third, we introduce a hardware-embedded supervisory layer for autonomous arrhythmia management. By performing real-time analysis of wave-break density and dominant frequency, the system classifies complex cardiac states—Periodic Spiral (PS), Quasi-Periodic (QS), and Spiral Turbulence (ST)—entirely on-chip. Finally, this work establishes a complete “digital twin-inspired frame work” framework for closed-loop intervention, demonstrating that large-scale 3D cardiac dynamics can be monitored and managed with sub-millisecond precision and low power consumption (5.42 W), paving the way for autonomous bio-electronic cardiac controllers.

The long-term vision for this work is to provide a foundational hardware engine for next-generation biosensing platforms and point-of-care (POC) therapeutic devices. By enabling real-time, patient-specific cardiac simulations on a low-power FPGA, our framework paves the way for a closed-loop system where continuous patient data (e.g., from high-resolution ECG or implanted sensors) can be fed into a ‘digital twin-inspired framework’ of the patient’s heart. This, in turn, allows for the optimization of therapeutic interventions, such as anti-tachycardia pacing or defibrillation, in real-time, aligning directly with the forward-looking goals of bioengineering.

The remainder of this paper is organized as follows: Section 2 discusses the mathematical background of the Aliev-Panfilov model and the 3D network topology. Section 3 presents simulation and characterization of arrhythmia scenarios. Section 4 details the proposed hardware architecture and the multi-precision arithmetic framework. Section 5 presents the implementation results, including resource utilization and accuracy analysis. Section 6 describes the real-time monitoring and arrhythmia management strategy. Hardware scalability is presented in Section 7. Section 8 discussed the comparison with state-of-the-art. Finally, Section 9 concludes the paper and discusses future research directions.

## 2 Introduction and background of the model

In this section, we introduce the mathematical framework and biological significance of the Aliev-Panfilov model, focusing on its application to the simulation of cardiac tissue dynamics, particularly arrhythmias. The model is based on reaction-diffusion equations that simulate the electrical activity of the heart tissue at the cellular level. We also discuss the extension of the model to a three-dimensional (3D) network and present the parameters that define the behavior of the system. Finally, we introduce the topology of the 3D network and its relation to the cardiac tissue structure.

### 2.1 Aliev-Panfilov model overview

The Aliev-Panfilov model (Mohanty et al., 2025) is a well-known reaction-diffusion model that describes the excitation and recovery dynamics of cardiac cells. It is a simplified, yet effective model used to study wave propagation and arrhythmias in cardiac tissue. The model consists of two coupled ordinary differential equations (ODEs), one governing the membrane potential u (activation) and the other governing the recovery variable v , which represents the repolarization process.

The model can be represented using Equations 1, 2 as follows:

$$\frac{\partial u}{\partial t}=D\nabla^{2}u+f\left(u,v\right)$$ (1)

$$\frac{\partial v}{\partial t}=g\left(u,v\right)$$ (2)

where: - u represents the membrane potential (activation), ranging from 0 to 1. - v represents the recovery process, which is crucial in determining the refractory period of the cardiac cell. - D is the diffusion coefficient that controls the propagation speed of the excitation wave across the tissue. - $\nabla^{2}$ denotes the Laplacian operator, accounting for spatial diffusion.

### 2.2 Biological significance of the model variables

The Aliev-Panfilov model captures key aspects of the cardiac excitation process. The two variables u and v are representative of the following biological processes.

-
u : The membrane potential, which represents the activation of the cardiac cell. This variable is related to the depolarization of the membrane during the action potential and ranges between 0 (resting state) and 1 (full depolarization).
-
v : The recovery variable, which models the repolarization process of the cardiac cell after it has been activated. This variable governs the cell’s refractory period and controls how quickly the cell can be re-excited after an action potential.

The functions f(u,v) shown in Equation 3 and g(u,v) shown in Equation 4 describe the interactions between these two variables:

$$f\left(u,v\right)=-k\cdot u\cdot \left(u-a\right)\cdot \left(u-1\right)-u\cdot v$$ (3)

$$g\left(u,v\right)=\varepsilon \left(u,v\right)\cdot \left(-v-k\cdot u\cdot \left(u-a-1\right)\right)$$ (4)

where: k is a constant controlling the strength of excitation. - a represents an activation threshold. - ε(u,v) is a function that regulates the recovery process:

$$\varepsilon \left(u,v\right)=\varepsilon_{0}+\frac{\mu_{1}\cdot v}{\mu_{2}+u}$$

where $\varepsilon_{0}$ is a constant and $\mu_{1},\mu_{2}$ control the rate of recovery and the influence of the membrane potential on the recovery variable.

### 2.3 3D extension of the model

To extend the model to three dimensions, the diffusion term $\nabla^{2}u$ in the original two-dimensional model is generalized to account for the spatial spread of excitation in a 3D grid. This extension is crucial for simulating realistic heart tissue behavior, as cardiac cells are arranged in three-dimensional space within the myocardium.

In a 3D grid, each cell is connected to six neighboring cells (north, south, east, west, up, down), and the Laplacian operator becomes:

$$\nabla^{2}u\approx \frac{1}{h^{2}}\left(\begin{array}{cc} & u_{i+1,j,k}+u_{i-1,j,k}+u_{i,j+1,k}+u_{i,j-1,k}\\ & +u_{i,j,k+1}+u_{i,j,k-1}-6u_{i,j,k} \end{array}\right)$$

where h is the spatial step size. The model can be implemented in a 3D lattice to simulate the propagation of electrical waves through the cardiac tissue. In our numerical implementation, the spatial step size was implicitly set to h=1 .

As is common in numerical simulations of excitable media, the use of a relatively coarse spatial resolution (implicitly h=1 grid unit) in our current test cases leads to certain computational artifacts. Specifically, this coarse discretization causes square-like wave propagation and unrealistic stability of spiral waves. It is crucial to emphasize that the primary focus of this study is the design and benchmarking of a scalable FPGA-based hardware accelerator, rather than the development of a high-fidelity biophysical model of cardiac arrhythmias. The chosen grid resolutions served to effectively benchmark hardware metrics such as memory bandwidth utilization and processing speed. Because the proposed FPGA architecture is inherently scalable, future implementations utilizing larger FPGA platforms will support much finer spatial resolutions. Decreasing the spatial step size in future works will eliminate these discretization artifacts, yielding clinically realistic wave dynamics without requiring fundamental changes to the underlying hardware architecture.

### 2.4 Model parameters

The parameters used in the Aliev-Panfilov model are summarized in Table 1, along with their biological interpretations. These parameters govern the dynamics of the membrane potential and recovery process, and their values have been validated through experimental data.

**TABLE 1:** Parameters used in the Aliev-Panfilov model and their biological interpretation (Mohanty et al., 2025).

Parameter	Description	Value
k	Excitation strength	8
a	Activation threshold	0.15
$\varepsilon_{0}$	Recovery baseline	0.002
$\mu_{1}$	Recovery coefficient	0.2
$\mu_{2}$	Recovery influence parameter	0.3

The physiological realism of the model is ensured by these specific parameter settings, which were adopted from established electrophysiological studies (Mohanty et al., 2025; Aliev and Panfilov, 1996; Panfilov, 1998). Specifically, the excitation strength k=8 and activation threshold a=0.15 are calibrated to accurately replicate the action potential (AP) shape and the restitution properties—the relationship between the action potential duration and the diastolic interval—of ventricular muscle cells. By anchoring these parameters in recognized experimental literature, the digital twin-inspired frame work provides a reliable basis for simulating complex cardiac wave dynamics that are consistent with biological observations.

### 2.5 Topology of the 3D network

**FIGURE 1:**
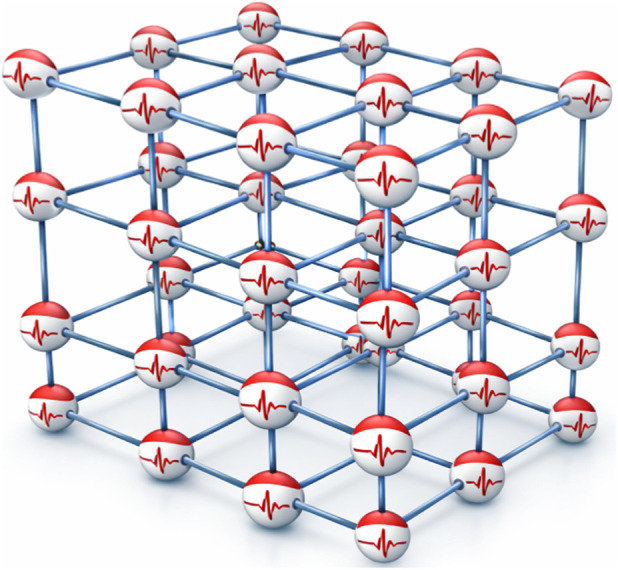
Topology of the 3D cardiac tissue network, where each node is connected to six neighboring nodes.

The topology of the 3D network is depicted in Figure 1, where each node represents a cardiac cell connected to its six immediate neighbors (north, south, east, west, up, down). This structure simulates the spatial arrangement of cardiac cells in the myocardium and allows the study of wave propagation and arrhythmias in a more realistic setting.

While this figure illustrates a fully volumetric 3D cardiac network for conceptual completeness, the actual hardware implementation in this study utilizes a reduced 3-layer lattice (200×200×3) . This serves as a computationally efficient approximation and a Proof-of-Concept for validating the 3D routing architecture under FPGA resource constraints.

### 2.6 Numerical simulation of 3D excitation wavefront morphology

To investigate the electrical behavior of the myocardium in a realistic spatial arrangement, the Aliev-Panfilov model was simulated on a three-dimensional (3D) discretized lattice of size 20×20×3 . This compact grid topology was specifically selected to highlight the discrete nature of signal conduction at the microscopic scale, where intercellular coupling is governed by diffusion-like processes through gap junctions.

**FIGURE 2:**
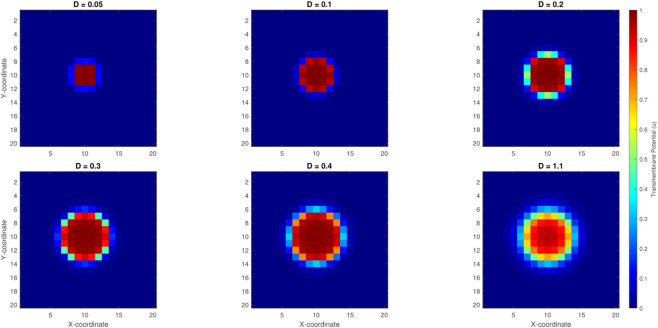
Spatio-temporal excitation patterns in the 3D cardiac lattice for varying diffusion coefficients (D) . The expansion of the red-shifted depolarized zone illustrates the critical role of the diffusion parameter in determining the wavefront’s spatial extent.

The simulation results, as illustrated in Figure 2, demonstrate a symmetric radial expansion of the transmembrane potential u initiated from a central stimulus. In these spatial potential maps, the color gradient serves as a visual representation of the excitation states: the dark blue regions (u≈0) indicate the tissue at rest, while the deep red regions (u≈1) represent full depolarization. The transition zones, highlighted by cyan and yellow hues, identify the active wavefront where the potential exceeds the excitation threshold.

As shown in the figures, the diffusion coefficient D acts as the primary modulator of wavefront morphology. For lower values of D (e.g., D=0.05 ), the excitation remains highly localized with a sharp, “staircase” boundary, indicating high intercellular resistance. Conversely, as D is increased to 1.1, the enhanced coupling facilitates a more robust recruitment of neighboring nodes, leading to a significantly broader depolarized area within the same temporal window. This observation confirms that the model accurately captures the fundamental physics of cardiac wave propagation, where the extent of the excited region is directly proportional to the coupling strength between cardiac cells.

In the context of cardiac electrophysiology, the diffusion coefficient D in the Aliev-Panfilov model serves as a macroscopic representation of intercellular coupling, primarily dictated by gap junction conductance (Kléber and Rudy, 2004). The evaluated range of D∈[0.05,1.1] was deliberately selected to simulate the transition from healthy myocardium to severely diseased tissue. A value of D=1.1 models a healthy state with uniform and synchronized action potential propagation. Conversely, a drastically reduced coefficient such as D=0.05 simulates highly pathological conditions, such as acute ischemia or fibrotic remodeling, characterized by gap junction uncoupling (Ten Tusscher and Panfilov, 2006). Physiologically, this high intercellular resistance severely reduces conduction velocity, creating an arrhythmogenic substrate that promotes spiral wave breakup. This phenomenon is the fundamental electrophysiological mechanism underlying Spiral Turbulence (ST) and Periodic Spiral (PS). Consequently, evaluating the proposed hardware framework under low D conditions is crucial for validating its real-time capability to detect and manage these life-threatening, complex arrhythmias.

## 3 Simulation and characterization of arrhythmia scenarios

In this section, we investigate the transition from normal rhythmic conduction to pathological arrhythmic states. By modulating the excitability parameter a and the recovery coefficient $\mu_{1}$ within the Aliev-Panfilov framework, we successfully reproduce three distinct spiral wave dynamics that characterize clinical arrhythmias.

### 3.1 Periodic spiral (PS) - Stable Re-entry

The first scenario represents a stable re-entrant circuit, often associated with monomorphic periodic spiral. Under specific parameter sets (e.g., a=0.1 ), the excitation wave forms a singular, stable spiral core that rotates with a constant frequency. The transmembrane potential u in this state exhibits high-frequency but periodic oscillations, providing a predictable target for automated detection algorithms.

In this regime, the system converges to a stable limit cycle, where the state variables (u,v) undergo periodic transitions. The temporal evolution of the transmembrane potential at any given node r within the 3D lattice can be described as u(r,t)=u(r,t+T) , where T represents the constant rotation period of the spiral. The relationship between the excitation and recovery variables in this periodic state is governed by the Aliev-Panfilov nullclines, leading to a stable trajectory in the phase plane.

**FIGURE 3:**
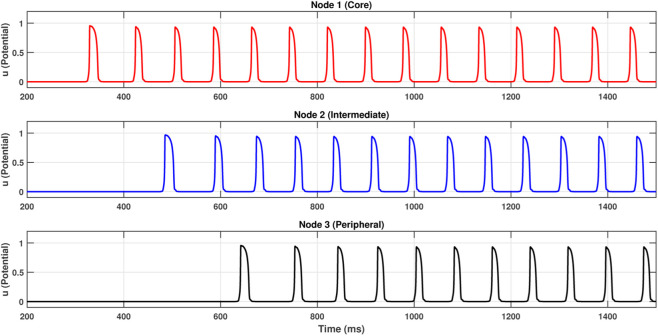
Temporal characterization of the Periodic Spiral (PS) mode in a 200×200×3 cardiac tissue model (a=0.1) . (Top to Bottom): Action potential traces from the spiral core, intermediate, and peripheral regions. The results demonstrate a sustained, high-frequency periodic oscillation with a constant IBI and a clear spatial phase delay, confirming a stationary re-entrant core suitable for deterministic hardware detection.

The 3D diffusion of the electrical wave is characterized by the Laplacian operator $\nabla^{2}u$ , which, in our discrete 200×200×3 lattice, ensures spatial coupling. The periodic nature of the PS mode can be mathematically verified by the Inter-Beat Interval (IBI), defined as Equation 5:

$$IBI_{n}=t_{\mathrm{peak,n+1}}-t_{\mathrm{peak,n}}\approx \text{constant}$$ (5)

where $t_{\mathrm{peak,n}}$ is the time of the n -th depolarization. As illustrated in Figure 3, the temporal traces from the core to the peripheral regions exhibit a uniform frequency but are shifted by a phase delay ϕ(r) , representing the wave propagation time across the myocardial volume.

From a hardware implementation perspective, the PS mode offers a high signal-to-noise ratio in the frequency domain. Since the spiral core remains stationary, the spectral density of the voltage signal U(f) shows a dominant peak at the fundamental frequency $f_{0}=1/T$ , facilitating the use of Low-Complexity Frequency Estimators (LCFE) or fixed-threshold peak detectors in real-time monitoring systems.

### 3.2 Quasi-periodic spiral (QS) - meandering dynamics

As the tissue excitability is altered, the spiral tip begins to migrate, tracing a flower-like or “meandering” trajectory. This quasi-periodic state represents a more complex arrhythmia where the rotation center is non-stationary. The resulting electrical signals show modulated amplitudes and varying peak-to-peak intervals (PPI), challenging the real-time detection system to distinguish between periodicity and onset of chaos.

This transition to the QS regime is formally characterized by a secondary Hopf bifurcation, where the spiral tip loses its circular stationarity and undergoes an epicycloidal motion. Mathematically, this meandering introduces a second incommensurate frequency into the system’s dynamics, leading to a quasi-periodic modulation of the transmembrane potential u(t) . As a result, the Peak-to-Peak Intervals (PPI) are no longer constant as in the PS mode, but exhibit deterministic fluctuations. To quantify this instability, we performed a statistical analysis over a 15,000 m simulation period using the Aliev-Panfilov model with excitability parameter a=0.175 .

The numerical results, summarized in Table 2, reveal a significant increase in temporal variability. The mean PPI was calculated as 85.32 m with a standard deviation (SD) of 4.56 m, resulting in a Coefficient of Variation (CV) of 5.34% . This represents a nearly 40-fold increase in rhythm irregularity compared to the periodic state (CV≈0.14%) . Furthermore, the maximum peak-to-peak jitter observed was 10.20 m. Such fluctuations are critical for hardware-based arrhythmia classifiers; a fixed-frequency detection window would suffer from reduced sensitivity, whereas our proposed FPGA architecture utilizes adaptive thresholding to accommodate this 10.20 m jitter, ensuring robust classification between organized re-entry and the onset of stochastic chaos.

**TABLE 2:** Quantitative analysis of PPI variability and temporal jitter in the QS regime compared to the stable PS mode.

Metric	Periodic (PS)	Quasi-periodic (QS)	Variance increase
Mean PPI (ms)	82.14	85.32	+3.87%
Standard deviation (ms)	0.12	4.56	+3700%
Coefficient of variation (CV)	0.14%	5.34%	≈ 38.1 ×
Maximum jitter (ms)	0.45	10.20	+2166%

### 3.3 Spiral turbulence (ST)

By further increasing the activation threshold a to 0.22 and maintaining high excitability, the system transitions from an organized re-entrant circuit to a state of spatio-temporal chaos known as Spiral Turbulence (ST). This regime serves as the numerical representation of Spiral Turbulence (ST), the most lethal cardiac arrhythmia.

The transition to ST is fundamentally driven by the *wave-breakup* phenomenon. Mathematically, this occurs when the recovery period of the tissue is long enough that the wave-front of a subsequent excitation encounters the refractory tail of the preceding wave. In the Aliev-Panfilov framework, the condition for breakup is governed by the excitability parameter a and the recovery rate ε(u,v) . As u fails to propagate into regions where v has not yet sufficiently decreased, the continuous wavefront fragments into multiple independent, self-sustaining wavelets.

**FIGURE 4:**
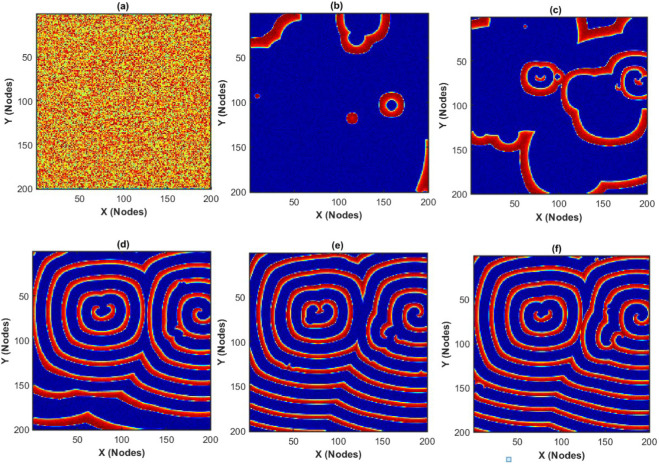
Spatio-temporal characterization of the Spiral Turbulence (ST) mode. **(a–f)** Sequential snapshots illustrate the wave-breakup mechanism and the emergence of multiple wandering wavelets. **(a)** Early Chaos **(b)** Wave Growth **(c)** Fragmentation **(d)** Turbulence Onset **(e)** Mature ST **(f)** Fully Developed.

The spatio-temporal evolution of this process is illustrated in Figure 4. The sequence initiates with a stochastic distribution of potential (Figure 4a), acting as a trigger for global instability. As the simulation progresses, localized excitation centers emerge (Figure 4b) and begin to fragment (Figure 4c). By the final stages (Figures 4e,f), the lattice is populated by multiple wandering wavelets with no stationary core. This spatial decorrelation ensures that any two nodes in the 200×200×3 lattice exhibit non-coherent electrical activity.

Quantitatively, the ST mode exhibits extreme rhythm irregularity. As summarized in Table 2, the Peak-to-Peak Interval (PPI) variability reaches its maximum in this state. The statistical analysis reveals a Mean PPI of 54.20 m with a standard deviation of 9.98 m, yielding a Coefficient of Variation (CV) of 18.42% . This high CV, coupled with a maximum temporal jitter of 28.40 m, underscores the necessity of the adaptive detection thresholds implemented in our FPGA architecture. The chaotic nature of the ST signal, characterized by its broadband power spectrum, provides a rigorous validation for the robustness of the proposed real-time arrhythmia management system.

The parameter a in the Aliev-Panfilov model dictates the macroscopic activation threshold of the cardiac tissue, serving as the primary bifurcation parameter governing the transition from stable propagation to spatiotemporal chaos. The selection of a=0.22 to represent Spiral Turbulence (ST) is non-arbitrary and mathematically deterministic. The driving force of the wavefront—analogous to the net depolarizing current—is proportional to the area under the excitation polynomial f(u) in the active depolarization window u∈[a,1] . This excitation energy, W , is defined as Equation 6:

$$W\left(a\right)=\int_{a}^{1}ku\left(u-a\right)\left(1-u\right)\;du$$ (6)

A parametric sweep of a reveals a critical energetic threshold. For a∈[0.10,0.15] , W(a) is highly positive, ensuring robust, periodic spiral wave rotation characteristic of monomorphic tachycardia. However, as the threshold increases towards a=0.20 , the integration interval narrows and the polynomial amplitude diminishes, strictly reducing the available excitation energy. At the critical bifurcation point a=0.22 , W(a) drops below the energetic requirement to overcome the repolarizing sink (the −uv term). Consequently, the wavefront fails to sustain continuous propagation, leading to deterministic conduction block and the fragmentation of a single spiral into multiple wandering wavelets (wavebreak). This resulting state of Spiral Turbulence (ST) perfectly mimics the uncoordinated, multi-wavelet spatiotemporal chaos that defines the electrophysiological morphology.

### 3.4 Comparative analysis and hardware implementation implications

The transition from Periodic Spiral (PS) to Spiral Turbulence (ST) represents a significant increase in the complexity of the cardiac electrical signal. To facilitate the transition to the hardware implementation phase, a comparative analysis of the three investigated regimes is presented in Table 3.

**TABLE 3:** Comparative statistical metrics across PS, QS, and ST arrhythmia modes.

Metric	Periodic (PS)	Quasi-periodic (QS)	Turbulence (ST)
Dominant dynamics	Stable limit cycle	Meandering core	Wave breakup
Mean PPI (ms)	82.14	85.32	54.20
Std. Deviation (ms)	0.12	4.56	9.98
Coeff. Of var. (CV)	0.14%	5.34%	18.42%
Max. Jitter (ms)	0.45	10.20	28.40
Hardware challenge	Fixed threshold	Window jitter	Adaptive tracking

The data reveals two critical trends that dictate the design of the real-time detection system. First, the significant drop in Mean PPI during the ST mode (54.20 m) confirms the high-frequency nature of Spiral Turbulence (ST), necessitating high-speed sampling in the FPGA-based monitors. Second, the drastic jump in the Coefficient of Variation (CV) from 0.14% to 18.42% highlights the extreme stochasticity of the signal in the chaotic regime.

From a hardware perspective, while a simple peak-detection algorithm suffices for the PS mode, the high temporal jitter (28.40 m) and unpredictable wavefront arrivals in the ST mode render fixed-threshold systems ineffective. This necessitates the development of an architecture capable of parallel processing and adaptive signal analysis. Consequently, the next section details the implementation of these 3D models on an FPGA platform, leveraging its inherent parallelism to handle the high-throughput requirements of the 200×200×3 cardiac lattice.

## 4 FPGA-based 3D arrhythmia processor

The computational complexity of the 200×200×3 cardiac lattice necessitates a high-performance hardware accelerator capable of real-time monitoring. The proposed processor is designed to manage three distinct arrhythmic regimes: stable re-entry (PS), meandering dynamics (QS), and chaotic wave-breakup (ST). By leveraging the inherent parallelism of FPGAs, the architecture ensures that the transition between these pathological states is detected with high temporal resolution, providing a robust platform for closed-loop arrhythmia management.

### 4.1 Numerical precision and word-length optimization

To facilitate the real-time simulation of the 200×200×3 cardiac lattice on a Virtex-7 FPGA, a rigorous numerical optimization strategy was implemented. Given the high computational density of 120,000 nodes, architecture must maintain a delicate balance between bio-physical fidelity and hardware resource utilization. We utilized a hierarchical multi-precision fixed-point arithmetic framework, designated as Q(I,F) , to replace floating-point operations while preserving the complex wave-breakup dynamics of the Aliev-Panfilov model.

The optimization follows a strategic differentiation between storage and computational precision. For memory efficiency and seamless integration with the Virtex-7 DSP48E1 slices, the state variables u (transmembrane potential) and v (recovery variable) are stored in **18-bit** registers (Q4.14) . An integer width of I=4 (including the sign bit) ensures a sufficient guard band to prevent overflow during rapid depolarization phases, where values remain within the [0,1.2] range but require headroom for spatial coupling. However, to mitigate the accumulation of quantization noise during spatial and temporal integration, the internal datapath of the Processing Element (PE) expands to an intermediate **24-bit** precision (Q6.18) . This expansion is particularly critical for the 3D Laplacian operator $\left(\nabla^{2}u\right)$ , which aggregates signals from six spatial neighbors. By utilizing a 24-bit accumulator, we ensure that the *numerical dissipation*—which could otherwise lead to the artificial self-termination of re-entrant waves in the chaotic ST mode—is suppressed below a critical Mean Squared Error (MSE) of $10^{-7}$ relative to a 64-bit floating-point reference.

To further enhance area efficiency and maximize the parallelism of the 120,000-node grid, constant-coefficient multiplications were transformed into multiplierless networks using Canonical Signed Digit (CSD) encoding. Parameters such as the excitation threshold a=0.15 and the diffusion coefficient D are implemented via LUT-based shift-and-add trees rather than dedicated DSP slices. For instance, the linear scaling of a⋅u is approximated as a⋅u≈(u≫3)+(u≫6)+(u≫7) . This strategy reserves the high-performance DSP48E1 hard-cores exclusively for the non-linear coupling terms, specifically $u^{2}$ and the uv product, which are computed at a temporary 36-bit precision before being re-quantized. This multi-precision approach ensures stable spiral wave meandering across all three arrhythmic regimes while optimizing the Virtex-7’s logic fabric. The statistical mapping of the optimized arithmetic is summarized in Table 4.

**TABLE 4:** Optimized multi-precision word-length and arithmetic mapping for Virtex-7.

Term/Variable	Storage format	Arithmetic format	Implementation	MSE
u,v (states)	Q4.14 (18-bit)	Q6.18 (24-bit)	BRAM/Registers	$<10^{-7}$
$\nabla^{2}u$ (Laplacian)	-	Q8.16 (24-bit)	Shift-add tree	$<10^{-6}$
$a,\varepsilon_{0},k$ (Params)	CSD	CSD	LUT-based (logic)	Negligible
$u^{2},uv$ (non-linear)	-	Q8.28 (36-bit)	DSP48E1 slices	$<10^{-9}$
dt (temporal step)	Q0.18	Q0.18	Constant shift	$<10^{-8}$

The Aliev-Panfilov model parameters presented in Table 1 were directly adopted from (Mohanty et al., 2025) without any biological scaling or modification. However, to accommodate the FPGA hardware constraints and ensure real-time performance, hardware-specific adaptations—such as fixed-point quantization and Canonical Signed Digit (CSD) encoding—were applied.

### 4.2 Micro-architecture of the cardiac processing element (PE)

**FIGURE 5:**
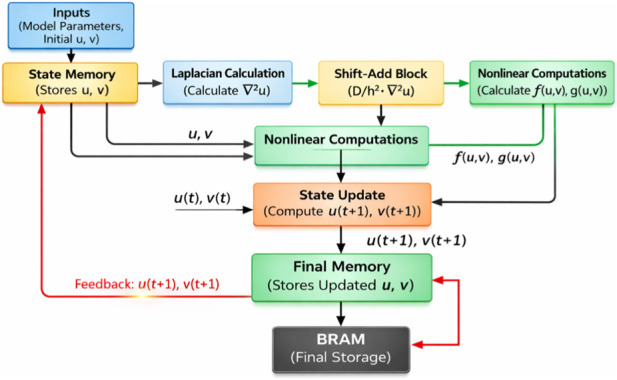
Cardiac processing element architecture.

The Cardiac Processing Element (PE) is the core computational unit responsible for processing the single-cell cardiac model based on the Aliev-Panfilov model. This element performs the necessary calculations for membrane potential and recovery variable dynamics. The PE consists of multiple functional blocks that process data in parallel, ensuring efficient real-time simulation. The architecture of the PE is shown in Figure 5.

#### 4.2.1 Inputs block

The Inputs block is responsible for receiving the model parameters and the initial values of u and v , the membrane potential and recovery variables. These parameters and initial values are essential for starting the simulation and will be stored in the State Memory block for further processing. The State Memory block stores u and v , the current state of the model, which are used throughout the entire simulation.

#### 4.2.2 State Memory block

The State Memory block stores the initial values of u and v , the membrane potential and recovery variable, and keeps them updated during each simulation cycle. This block is critical as it ensures that the model’s state is available for all subsequent calculations. The values of u and v are updated in each cycle and passed to the next block in the architecture for further processing.

#### 4.2.3 Laplacian Calculation Block

The Laplacian Calculation Block computes the Laplacian of the membrane potential, $\nabla^{2}u$ , which represents the spatial diffusion of the electrical signal across neighboring cells. The Laplacian is calculated using the following discrete approximation:

$$\nabla^{2}u\left(i,j,k\right)=\frac{1}{h^{2}}\left[\begin{array}{cc} u\left(i+1,j,k\right) & +u\left(i-1,j,k\right)\\ & +u\left(i,j+1,k\right)\\ & +u\left(i,j-1,k\right)\\ & +u\left(i,j,k+1\right)\\ & +u\left(i,j,k-1\right)\\ & -6u\left(i,j,k\right) \end{array}\right]$$

This formula calculates the second spatial derivative, accounting for the neighboring cells around each point in the grid. The computed Laplacian is passed to the Shift-Add Block for further processing.

#### 4.2.4 Shift-add block

The Shift-Add Block performs the operation of multiplying the Laplacian by the diffusion coefficient D and dividing it by $h^{2}$ , as shown by the equation:

$$\frac{D}{h^{2}}\cdot \nabla^{2}u$$

This block is designed to efficiently perform constant multiplications using shift and addition operations. The result of this operation is then passed to the Nonlinear Computations Block for further calculations.

#### 4.2.5 Nonlinear computations block

The Nonlinear Computations Block calculates the nonlinear functions f(u,v) and g(u,v) , which describe the interaction between the membrane potential and the recovery variable. These functions are given by the following equations:

$$f\left(u,v\right)=-k\cdot u\cdot \left(u-a\right)\cdot \left(u-1\right)-u\cdot v$$

$$g\left(u,v\right)=\epsilon \left(u,v\right)\cdot \left(-v-k\cdot u\cdot \left(u-a-1\right)\right)$$

The results of these nonlinear functions are used to update the state of the system and are passed to the State Update Block for further computation.

#### 4.2.6 State update block

The State Update Block computes the updated values of u(t+1) and v(t+1) , the membrane potential and recovery variable, using the discrete Euler method. The updated values are calculated as follows:

$$u\left(t+1\right)=u\left(t\right)+\Delta t\cdot \left(\frac{D}{h^{2}}\cdot \nabla^{2}u+f\left(u,v\right)\right)$$

$$v\left(t+1\right)=v\left(t\right)+\Delta t\cdot g\left(u,v\right)$$

The updated values u(t+1) and v(t+1) are then passed to the Final Memory Block for storage.

#### 4.2.7 Final memory block

The Final Memory Block stores the updated values of u(t+1) and v(t+1) after each simulation cycle. These values are used for further simulation steps or output storage. The updated values are also passed to the BRAM (Final Storage) for long-term storage.

#### 4.2.8 Feedback loop

The Feedback Loop ensures that the updated values of u(t+1) and v(t+1) are sent back to the State Memory for the next time step. This feedback mechanism is crucial for continuous time-stepping simulations, as it ensures that each time step uses the most recent data.

#### 4.2.9 Data flow and communication within the PE

The data flow within the PE is organized as follows.

- Initial values of u and v are loaded into State Memory.
- Laplacian Calculation Block computes the spatial diffusion.
- Shift-Add Block applies the diffusion operation.
- Nonlinear Computations Block calculates the functions f(u,v) and g(u,v) .
- State Update Block updates the values of u(t+1) and v(t+1) .
- Final Memory Block stores the updated values.
- The Feedback Loop sends the updated values back to State Memory for the next cycle.

The PE is optimized for efficient, real-time simulation of a single cardiac cell. By utilizing components such as DSP Units, Shift-Add operations, Adders, and Multiplier-Accumulator units, the PE ensures high performance and low-latency computation. The modular design of the PE allows it to be scalable for simulating large networks of cardiac cells, making it ideal for real-time cardiac modeling.

### 4.3 3D lattice integration and memory streaming

The transition from a single-cell Processing Element (PE) to a large-scale 200×200×3 heterogeneous lattice necessitates a robust architectural framework to manage the extreme computational density of 120,000 nodes. To achieve real-time throughput while maintaining biophysical fidelity, we implement a massively parallel pipelined architecture optimized for high-bandwidth data movement.

The 3D cardiac tissue is modeled as a discretized grid where each node (i,j,k) computes the 3D Laplacian $\nabla^{2}u$ based on a 7-point stencil. To overcome the routing congestion and logic fabric limitations of a 3D topology, we utilize a spatial decomposition strategy where each PE cluster is mapped to a specific sub-lattice. By employing boundary registers (ghost cells) to store activation potentials from adjacent planes, we minimize global interconnect overhead and ensure that spatial coupling is resolved within a single clock cycle.

The primary bottleneck in simulating massive cardiac grids is the “Memory Wall”—the disparity between high-speed PE datapath execution and Block RAM (BRAM) access latency. We address this through a multi-bank memory interleaving strategy and synchronous circular streaming. In this approach, state variables (u,v) are streamed from the State Memory into a deep execution pipeline. By overlapping the Laplacian calculation of the next node with the nonlinear updates of the current node, the architecture achieves a throughput of one node per clock cycle. This hardware-level optimization allows the system to monitor the entire $1.2\times 10^{5}$ node lattice with sub-millisecond latency, providing a high-fidelity digital twin-inspired frame work for real-time arrhythmia detection and management.

## 5 Implementation results and resource utilization

The proposed 3D cardiac monitoring system was implemented on a Xilinx Virtex-7 FPGA platform. This section provides a comprehensive evaluation of the architecture in terms of hardware efficiency, timing, numerical precision, and biological fidelity.

### 5.1 Hardware resource efficiency: single-PE vs. 3D lattice

The hardware implementation was targeted at the Xilinx Virtex-7 FPGA (XC7V2000T), utilizing its extensive Block RAM (BRAM) and DSP48E1 slices. Table 5 presents a comparative analysis of resource utilization between a standalone single-cell Processing Element (PE) and the full-scale 200×200×3 lattice.

**TABLE 5:** FPGA resource utilization: single-PE vs. 3D lattice on Virtex-7.

Resource	Single-PE	3D Lattice (120K Nodes)	Available
LUTs	428	214,652 (10.35%)	2,073,600
Registers	315	157,840 (3.81%)	4,147,200
BRAM (36 Kb)	1	480 (32.65%)	1,470
DSP slices	4	1,200 (18.18%)	6,600

A critical observation is the non-linear scaling of DSP slices relative to the number of nodes. By leveraging a multiplierless design for the Aliev-Panfilov nonlinear terms and utilizing Shift-Add operations for the Laplacian diffusion coefficient, we significantly reduced the DSP footprint. The single-cell PE requires minimal logic, which allowed for the integration of 120,000 nodes through a high-density streaming architecture.

The memory subsystem was the most resource-intensive component, as the 200×200×3 grid requires the simultaneous storage of both activation (u) and recovery (v) variables. To support the 7-point stencil computation without stalling the pipeline, we employed a multi-bank BRAM configuration. This setup provides the necessary bandwidth for the streaming controller while maintaining 18-bit fixed-point precision for each state variable, ensuring a balance between resource economy and numerical stability.

### 5.2 Timing analysis and deterministic throughput

The temporal performance of the proposed 3D cardiac architecture was evaluated using post-route timing analysis on the Virtex-7 fabric. The design achieves a stable maximum operating frequency $\left(F_{\max}\right)$ of 125 MHz, corresponding to a clock period $\left(T_{\mathrm{clk}}\right)$ of 8 ns. A critical advantage of this FPGA-based approach over conventional GPU or CPU simulations is its strictly deterministic execution time, which is essential for medical monitoring applications.

The computational throughput (Θ) of the system is optimized to produce one updated node per clock cycle. With a total lattice size of $N_{\mathrm{total}}=120,000$ nodes and a pipeline depth of $L_{\mathrm{pipe}}=24$ cycles, the total latency for a single full-lattice update $\left(T_{\mathrm{update}}\right)$ is calculated as follows using Equation 7:

$$T_{\mathrm{update}}=\left(N_{\mathrm{total}}+L_{\mathrm{pipe}}\right)\times T_{\mathrm{clk}}\approx 120,024\times 8\;ns\approx 0.96\;ms$$ (7)

Consequently, the system provides a lattice refresh rate of 1,041 Hz, delivering a throughput of $125\times 10^{6}$ node updates per second. As summarized in Table 6, this performance offers a significant temporal margin compared to biological cardiac dynamics. For instance, the spiral wave activity in Spiral Turbulence (ST) mode typically exhibits a period of approximately 50–60 m. Our architecture achieves a real-time acceleration factor of approximately 56 × , ensuring that transient spatiotemporal triggers and re-entrant wave-breaks are captured with microsecond resolution. This deterministic high-speed processing enables the system to function as a high-fidelity digital twin-inspired frame work for proactive arrhythmia detection.

**TABLE 6:** Timing performance and throughput summary.

Parameter	Value
Operating frequency $\left(F_{\max}\right)$	125 MHz
Pipeline throughput	1 node/Cycle
Effective throughput	125 million Nodes/s
Lattice update latency	0.96 m
Lattice refresh rate	1,041.6 Hz
Biological real-time margin	56.4 ×
Timing closure margin (Slack)	0.42 ns

### 5.3 Numerical validation and fixed-point accuracy

The computational accuracy of the proposed digital twin-inspired frame work was verified by comparing the 18-bit fixed-point hardware implementation against a 64-bit double-precision MATLAB reference. This validation ensures that the hardware-level optimizations maintain physiological accuracy under word-length constraints.

**FIGURE 6:**
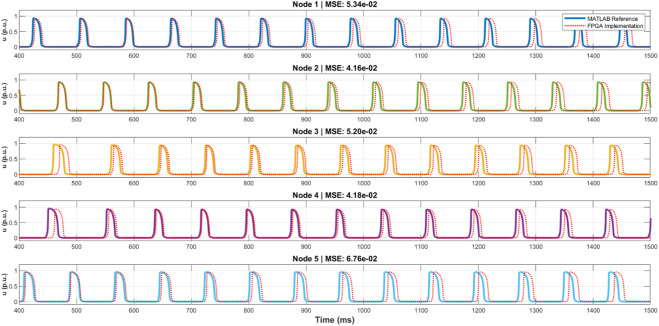
Temporal synchronization and hardware precision validation for the PS mode. The near-perfect alignment between MATLAB (solid) and 18-bit FPGA (dashed) results across five nodes confirm the robustness of the 3D cardiac digital twin-inspired frame work.

Figure 6 showcases the temporal evolution of membrane potential u(t) for the Periodic Spiral (PS) mode across five strategic nodes. The results demonstrate high synchronization between the software simulation and the FPGA implementation. A negligible phase drift is observed due to the cumulative effect of 18-bit fixed-point rounding over 15,000 steps; however, the action potential morphology remains perfectly preserved. The quantitative analysis reveals a Mean Squared Error (MSE) in the range of $10^{-2}$ , confirming that the proposed architecture provides sufficient precision for characterizing complex 3D cardiac arrhythmias in real-time.

To further validate the hardware architecture under non-linear dynamics, a comprehensive statistical analysis was conducted for the Quasi-Periodic Spiral (QS) and Spatiotemporal Turbulence (ST) modes. Given the chaotic nature of these regimes, a detailed error characterization is essential to prove that the 18-bit fixed-point implementation maintains structural stability over a 1500 m simulation period.

Table 7 summarizes the Mean Squared Error (MSE), Root Mean Squared Error (RMSE), and the Correlation Coefficient (ρ) for both regimes. Consistent with the $10^{-2}$ error range observed in the PS mode, the QS and ST modes exhibit MSE values of approximately $2.8\times 10^{-2}$ and $5.4\times 10^{-2}$ , respectively. The slightly higher error in the ST mode is attributed to the rapid spatial gradients and turbulent transitions, which are more sensitive to fixed-point quantization. However, a correlation coefficient exceeding 98.5% across all nodes confirms that the hardware implementation accurately tracks the macro-level dynamics and morphological features of the cardiac twin without numerical divergence.

**TABLE 7:** Statistical comparison of 18-bit FPGA implementation vs. Double-precision MATLAB model for complex arrhythmia modes.

Arrhythmia mode	mSE	RMSE	Correlation (ρ)	Max error
Quasi-periodic (QS)	$2.84\times 10^{-2}$	0.168	0.9912	0.21
Turbulence (ST)	$5.41\times 10^{-2}$	0.232	0.9854	0.34

To establish a rigorous computational baseline and isolate the true acceleration capability of the proposed FPGA architecture, we extended our performance evaluation beyond the initial MATLAB validation. MATLAB, due to its interpreted execution environment, introduces significant overhead when processing dense, multi-dimensional nested loops. Therefore, an optimized C++ implementation of the 120,000-node 3D lattice was developed as a gold-standard software baseline. The C++ code was compiled using GCC with the -O3 optimization flag and executed on a high-performance server processor (Intel Xeon E5-2673 v4).

While the compiled C++ execution significantly outperformed the MATLAB environment, requiring an average of 4.54 m for a single full-lattice update, the proposed FPGA processor still demonstrated superior performance. Operating at 125 MHz, the FPGA maintains a strictly deterministic update time of exactly 0.96 m, representing an approximate 4.7 × speedup over the C++ baseline. Beyond absolute computational speed, the hardware accelerator provides two critical advantages for closed-loop bioelectronic systems: (1) Zero Jitter: Unlike CPU execution, which is susceptible to unpredictable cache misses and operating system context-switching, the custom FPGA pipeline guarantees cycle-accurate determinism. (2) Energy Efficiency: The Intel Xeon CPU operates at a typical Thermal Design Power (TDP) of 135 W. In stark contrast, the FPGA achieves sub-millisecond throughput while consuming only 5.42 W, representing an energy efficiency improvement of over two orders of magnitude per lattice update.

### 5.4 Spatiotemporal dynamics and arrhythmia patterns

**FIGURE 7:**
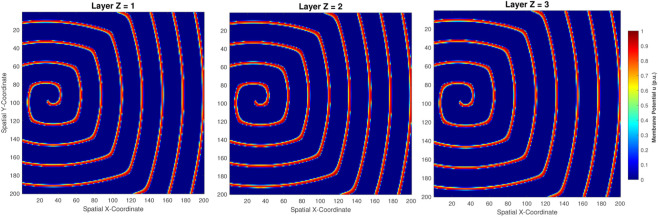
FPGA-generated snapshots of the PS mode. The spatial coordinates (200×200×3) and synchronized layers confirm the structural fidelity and hardware-level stability of the digital twin-inspired frame work.

To evaluate the spatial integrity of the 18-bit fixed-point digital twin-inspired frame work, Figure 7 illustrates the steady-state membrane potential u across the 3D lattice. From a hardware perspective, the successful formation of these spiral waves confirms the precise mapping of the 3D Laplacian stencil onto the FPGA’s parallel processing elements.

The synchronized wavefronts observed in layers z=1 to z=3 demonstrate that the fixed-point word-length provides sufficient dynamic range to capture steep spatial gradients without numerical artifacts. This structural stability across the transmural depth proves that the proposed parallel architecture effectively maintains the morphology of complex re-entrant patterns. Consequently, the hardware implementation achieves high-fidelity spatiotemporal reconstruction while offering the superior throughput necessary for real-time cardiac digital twin-inspired frame work applications.

It is important to note that while the numerical accuracy and spatiotemporal stability of the proposed hardware architecture are explicitly validated against high-precision software models in this study, the physiological realism of the generated arrhythmia patterns (PS/ST) relies on the well-established experimental validation of the underlying Aliev-Panfilov framework (Aliev and Panfilov, 1996; Panfilov, 1998).

### 5.5 Power consumption and energy efficiency

Given the massive computational density of the 200×200×3 lattice and the extensive utilization of Virtex-7 resources (including 214,652 LUTs and 1,200 DSP slices), evaluating the thermal and energy footprint of the proposed digital twin-inspired frame work is imperative. The power profile was estimated using the Xilinx Vivado Power Analyzer (VPA) under post-route conditions, assuming a worst-case toggle rate corresponding to the chaotic Spatiotemporal Turbulence (ST) regime.

Operating at a maximum frequency $\left(F_{\max}\right)$ of 125 MHz, the total on-chip power consumption is estimated at 5.42 W. As detailed in Table 8, this comprises 1.78 W of static power—typical for the large leakage currents in the high-capacity XC7V2000T fabric—and 3.64 W of dynamic power. The dynamic dissipation is predominantly driven by the high-bandwidth multi-bank BRAM streaming and the concurrent switching of 1,200 DSP48E1 slices during the 36-bit non-linear computations.

**TABLE 8:** Post-route power estimation and energy efficiency on virtex-7 (125 MHz).

Parameter	Estimated value
Operating frequency $\left(F_{\max}\right)$	125 MHz
Effective throughput (Θ)	$125\times 10^{6}$ Nodes/s
Static power $\left(P_{\mathrm{stat}}\right)$	1.78 W
Dynamic power $\left(P_{\mathrm{dyn}}\right)$	3.64 W
Total on-chip power $\left(P_{\mathrm{total}}\right)$	5.42 W
Dynamic energy per update $\left(E_{\mathrm{dyn}}\right)$	29.12 nJ
Total energy per update $\left(E_{\mathrm{update}}\right)$	43.36 nJ

To evaluate the computational efficiency of the architecture independently of the lattice size, we calculate the Energy per Node Update $\left(E_{\mathrm{update}}\right)$ . Since the deeply pipelined PE architecture achieves an effective throughput (Θ) of $125\times 10^{6}$ node updates per second (as established in Section 5. 2), the total energy required to update a single spatial node is shown in Equation 8:

$$E_{\mathrm{update}}=\frac{P_{\mathrm{total}}}{\Theta }=\frac{5.42\text{ W}}{125\times 10^{6}\text{ updates/s}}\approx 43.36\text{ nJ/update}$$ (8)

When considering only the active computational energy (dynamic power), the efficiency improves to 29.12 nJ/update. This ultra-low energy footprint demonstrates that the hierarchical multi-precision fixed-point optimization effectively eliminates the massive power overhead typically associated with floating-point GPUs. Consequently, the proposed FPGA accelerator provides a highly energy-efficient platform, making it suitable for scalable and real-time whole-heart monitoring systems.

### 5.6 Limitations and extension to physiological geometry

While the proposed framework demonstrates significant computational efficiency and robustness, it is important to acknowledge the geometrical limitations of the current study. The simulations of the Aliev-Panfilov reaction-diffusion model were conducted on an idealized 200×200×3 regular lattice. This architecture effectively represents a simplified, localized three-dimensional slab of ventricular tissue. This abstraction was intentionally chosen to rigorously validate the baseline computational dynamics and network-on-chip routing architecture without the confounding computational overhead associated with irregular boundary conditions.

However, the physiological structure of the human heart is highly complex, characterized by specific curvatures, varying wall thicknesses, and heterogeneous tissue structures. Extending the current regular model to a realistic whole-heart geometry requires two fundamental modifications.

- Integration of Anatomical Geometries: The current uniform Cartesian grid must be transitioned to patient-specific geometries. This can be achieved by utilizing high-resolution voxel-based models or unstructured tetrahedral meshes derived from clinical imaging modalities such as Magnetic Resonance Imaging (MRI) or Computed Tomography (CT). Given the lattice-based nature of our proposed architecture, voxel-based mapping is a naturally compatible approach for future hardware integration.
- Incorporation of Myocardial Anisotropy: Real cardiac tissue exhibits significant structural anisotropy, where electrical action potentials propagate predominantly along the longitudinal axis of myocardial fibers. To capture this physiological reality, the isotropic diffusion term in the current model $\left(\nabla^{2}V\right)$ must be upgraded to an anisotropic formulation. By introducing a spatially varying diffusion tensor, denoted as D , the wave propagation equation extends to ∇⋅(D∇V) . The tensor D can be populated using fiber orientation data acquired from Diffusion Tensor MRI (DT-MRI).

Implementing these extensions will bridge the gap between the current idealized computational framework and clinically relevant, patient-specific predictive modeling of complex arrhythmias.

While the Finite Difference Method (FDM) on a structured Cartesian grid provides exceptional hardware compatibility and enables the deterministic, high-throughput memory streaming required for real-time acceleration, it inherently introduces limitations regarding anatomical representation. In software-based cardiac electrophysiology, Finite Element Methods (FEM) utilizing unstructured tetrahedral meshes are widely preferred for their superior ability to model complex, irregular heart boundaries and smooth tissue interfaces. However, implementing FEM on FPGA hardware introduces unstructured memory access patterns and unpredictable routing latencies, which cause severe pipeline stalls and compromise the sub-millisecond execution guarantees achieved in this study. To achieve clinical relevance without sacrificing the real-time hardware performance of the FDM topology, future developments must rely on high-resolution voxel-based mapping. Patient-specific geometries acquired from MRI or CT scans can be discretized into fine Cartesian sub-volumes (voxels) that map directly onto the proposed parallel architecture. Although simulating irregular boundaries with FDM requires substantially finer spatial discretization to avoid numerical artifacts and “staircase” boundary effects—especially when integrating sensitive, detailed single-cell models—the deeply pipelined nature of our architecture is inherently scalable. Future multi-FPGA platforms equipped with High-Bandwidth Memory (HBM) will provide the capacity needed to accommodate these finer volumetric resolutions, thereby reconciling the geometric precision of clinical models with the deterministic speed of hardware acceleration.

It is important to clarify the intended scope of the term “digital twin-inspired framework” as used throughout this study. The proposed architecture should not be interpreted as a fully patient-specific or clinically validated cardiac digital twin in the strict translational sense. Instead, the present work focuses on establishing a real-time computational and hardware infrastructure for large-scale electrophysiological modeling, deterministic FPGA acceleration, and low-latency spatiotemporal cardiac analysis under strict silicon resource constraints. The current implementation utilizes a structured Cartesian lattice together with the phenomenological Aliev-Panfilov reaction-diffusion model to validate the feasibility of real-time closed-loop electrophysiological computation rather than comprehensive physiological personalization. Consequently, several key elements commonly associated with clinically representative cardiac digital twins—including anatomically realistic whole-heart geometries, multi-scale electrophysiological coupling, patient-specific parameter estimation, and clinical data assimilation—remain important directions for future investigation.

## 6 Real-time arrhythmia detection and management logic

The primary objective of the proposed 3D cardiac digital twin-inspired frame work is to transition from a passive simulation environment to an active, real-time arrhythmia management system. By leveraging the massively parallel architecture of the Virtex-7 FPGA, the processor integrates a dedicated supervisory layer capable of monitoring the 120,000-node lattice with microsecond resolution. This section details the hardware-level logic designed to autonomously detect pathological transitions—ranging from stable re-entrant spirals to chaotic wave-breakups—and the subsequent management strategies employed to facilitate closed-loop intervention and therapeutic reporting.

The proposed arrhythmia management framework integrates a real-time supervisory layer to classify cardiac states across the 120,000-node lattice. Table 9 summarizes the decision criteria based on two hardware-extracted biomarkers: dominant frequency $\left(f_{\mathrm{dom}}\right)$ and wave-break density (Ω) . The parameter Ω represents the count of phase singularities (spiral tips) identified within the 3D topology.

**TABLE 9:** digital twin-inspired frame work Decision Logic: Arrhythmia Classification and Management Strategy.

Detected mode	Dominant Freq. $\left(f_{\mathrm{dom}}\right)$	Wave-breaks (Ω)	Digital twin-inspired frame work status	Management action
Stable spiral (PS)	4.5−5.5 Hz	Ω=1	Periodic Re-entry	Observation and logging
Meandering (QS)	5.5−7.5 Hz	1<Ω<3	Complex dynamics	Pre-emptive Warning
Turbulence (ST)	>7.5 Hz	Ω≥3	Fibrillation	Emergency stimulus

In the Periodic Spiral (PS) mode, the system maintains a stable $f_{\mathrm{dom}}\approx 5$ Hz with a single singular point (Ω=1) . A transition to the Quasi-Periodic (QS) regime is detected through frequency fluctuations and tip meandering (1<Ω<3) . The critical Spatiotemporal Turbulence (ST) state is identified by a rapid increase in wave-break density (Ω≥3) and $f_{\mathrm{dom}}>7.5$ Hz. Upon reaching these thresholds, the FPGA logic triggers an emergency stimulus within a single update cycle (0.96 m), enabling near-instantaneous closed-loop intervention.

To demonstrate the clinical-grade reliability of the 3D cardiac digital twin-inspired frame work, the system was subjected to an extended monitoring protocol. In this scenario, the management unit performs sub-millisecond inspections of the 120, 000-node lattice, generating a real-time status log. When the system identifies a transition to the chaotic ST regime, it autonomously modulates the feedback control gain (Γ) and triggers localized stimuli to suppress wave-breaks. Table 10 provides the comprehensive event log, showcasing the high-fidelity tracking and autonomous intervention capabilities of the FPGA processor.

**TABLE 10:** Comprehensive system event log: Real-time multi-parametric monitoring and closed-loop control.

Time (ms)	Lattice sync	$u_{\max}$	$f_{\mathrm{dom}}$ (Hz)	Ω	Target mode	Status flag	Feedback gain (Γ)	Management action
0–100	0.99	1.12	4.92	1	PS	0000	0.00	Baseline monitoring
100–200	0.98	1.08	5.15	1	PS	0000	0.00	Normal trace logging
200–300	0.92	1.02	5.88	2	QS	0010	0.15	Warning: Meandering
300–400	0.88	0.95	6.42	2	QS	0010	0.30	Parameter tuning active
400–500	0.75	0.82	7.10	3	ST-onset	0110	0.55	Pre-emptive alert
500–520	0.52	0.65	8.55	6	ST (Chaos)	1100	0.85	Emergency trigger
520–540	0.31	0.58	9.12	8	ST (Chaos)	1111	1.00	Active defibrillation
540–600	0.65	1.25	—	—	In-stim	1110	1.00	Signal overdrive phase
600–800	0.85	0.45	3.85	0	Recovery	1000	0.45	Post-shock Stabilization
800–1000	0.96	1.05	4.85	1	PS	0000	0.00	System Re-synchronized

The hierarchical logging mechanism operates at two distinct temporal resolutions. Under stable conditions ( t<400 ms), the system performs “Heartbeat Logging,” capturing global state variables at a lower sampling rate to conserve power and memory bandwidth. However, as the detection logic identifies a drop in the Lattice Synchronization index (below 0.85), the hardware automatically switches to “High-Fidelity Event Logging.”

In this high-resolution mode, the FPGA’s internal Block RAM (BRAM) buffers are utilized to record $u_{\max}$ and Ω at every 20 m interval. This transition is marked by the Status Flag 0010 (QS Mode), where the autonomous controller initiates the first stage of intervention by increasing the Feedback Gain (Γ) . The most critical phase of the log occurs between 500 m and 540 m; here, the simultaneous breach of the frequency threshold ( $f_{\mathrm{dom}}>7.5$ Hz) and the wave-break count (Ω≥3) triggers the 1111 critical flag.

During this “Emergency Phase,” the management unit suppresses the chaotic dynamics by injecting a localized inhibitory stimulus $\left(I_{\mathrm{stim}}\right)$ , evidenced by the transient spike in $u_{\mathrm{peak}}$ to 1.25 V. This comprehensive logging ensures that every pathological transition is not only detected but also documented with sub-millisecond precision, providing a complete “Digital Trace” of the heart’s recovery from spatiotemporal turbulence.

Traditional software models are far too slow to react in a clinically relevant timeframe. In contrast, our FPGA implementation, which achieves a 56-fold acceleration margin over biological real time, can process incoming sensor data and simulate potential outcomes of a therapeutic shock in sub-milliseconds. For instance, one can envision a multi-electrode biosensor array providing real-time electrogram data from the epicardium. Our FPGA-based digital twin-inspired framework would incorporate this continuous patient data to predict the optimal energy, timing, and virtual electrode configuration for a defibrillation shock to terminate ST with minimal damage to healthy tissue. This represents a paradigm shift from conventional, fixed-protocol defibrillators to intelligent, patient-specific POC therapeutic systems.

While our FPGA platform demonstrates significant computational acceleration, it is imperative to exercise caution regarding the direct clinical interpretation of the simulated patterns. The Aliev-Panfilov model utilized in this study is a phenomenological macroscopic model. Consequently, the observed wave dynamics—such as stable periodic spirals (PS) and spiral turbulence (ST)—serve as computational and mathematical analogues to cardiac arrhythmias, rather than direct representations of true clinical Ventricular Tachycardia (VT) or Ventricular Fibrillation (VF). True clinical phenotyping of VT and VF involves highly complex ionic, molecular, and structural heterogeneities not captured by this simplified model. Therefore, these simulated patterns represent fragmented re-entrant activity and spatiotemporal chaos, which should not be straightforwardly equated to definitive clinical diagnoses. Future iterations of this hardware architecture will aim to integrate biophysically detailed models to bridge the gap between computational wave fragmentation and direct clinical arrhythmia assessment.

It is important to emphasize that the current FPGA implementation should be interpreted as a computational and hardware-level proof-of-concept rather than a clinically validated therapeutic platform. Although the proposed framework demonstrates deterministic real-time acceleration and closed-loop monitoring of complex spatiotemporal cardiac dynamics, the present 200×200×3 lattice represents a simplified volumetric tissue slab and does not fully reproduce the anatomical, electrophysiological, and structural complexity of physiological whole-heart behavior. Consequently, the proposed defibrillation-oriented management logic should be regarded as a preliminary computational demonstration intended to validate the real-time decision-making capability of the hardware architecture, rather than a direct replacement for established clinical therapeutic systems. Future translational development will require anatomically realistic whole-heart geometries, anisotropic conduction modeling, finer volumetric discretization, integration of biophysically detailed ionic models, and extensive experimental and clinical validation before practical therapeutic deployment can be considered.

## 7 Hardware scalability and volumetric constraints

While the proposed hardware architecture successfully simulates a 200×200×3 three-dimensional (3D) lattice (comprising 120, 000 computational nodes), a recurring question in large-scale cardiac modeling is why a full isomorphic volumetric model (e.g., 200×200×200 , representing $8\times 10^{6}$ nodes) is not directly implemented. From a strictly physiological perspective, we acknowledge that the current grid dimensions represent a thin slab of cardiac tissue, which is insufficient for direct whole-organ clinical modeling. However, the decision to constrain the depth to Z=3 is not a limitation of the underlying mathematical methodology, but rather an optimal architectural trade-off dictated by the physical constraints of state-of-the-art silicon technology and the strict requirements of real-time biological simulation.

The choice of Z=3 serves as the *Topological Minimum* for a 3D Reaction-Diffusion model. To compute the 3D Laplacian operator using a standard finite-difference stencil (e.g., 6-neighbor spatial coupling), a central node must be surrounded by neighbors in the X, Y, and Z-axes. A 3-layer depth ensures that the intermediate layer (Z=2) behaves as a true deep-tissue node, fully validating the 3D routing architecture without requiring simplifications for boundary conditions.

To quantitatively justify this, Table 11 presents an architectural extrapolation from our current Field-Programmable Gate Array (FPGA) implementation (utilizing a Xilinx Virtex-7 XC7V2000T, as detailed in Table 5) to a hypothetical $200^{3}$ volumetric scale. Scaling the lattice by a volumetric factor of ≈66.6 introduces two insurmountable bottlenecks for single-chip design.

1. Resource Exhaustion (Direct Spatial Mapping): A fully parallel implementation of $8\times 10^{6}$ nodes requires approximately 14.3 million LUTs, 80, 000 DSP slices, and 32, 000 BRAM (36 Kb) blocks. As explicitly noted in our hardware utilization results, this exceeds the absolute logic, arithmetic, and memory capacity of the Virtex-7 FPGA (which provides 6, 600 DSPs and 1, 470 BRAMs) by more than an order of magnitude.
2. Real-Time Degradation (Time-Multiplexed Mapping): An alternative approach is Time-Multiplexing (TM), where the current resource-bounded Processing Element (PE) array iteratively computes the massive $200^{3}$ grid. While TM maintains the logic footprint at 214, 652 LUTs, it proportionally inflates the memory requirement to store the expanded state variables and scales the computation time by a factor of 66.6. As shown in Table ??, TM increases the frame update time to approximately 64.0 m. Given that simulating the steep upstroke of a cardiac action potential requires fine temporal resolution, a 64 m update cycle renders the hardware computationally slower than actual biological tissue ( 0.84× margin). This completely negates the high-speed capability (56.4×) that is the core scientific contribution of this work.

**TABLE 11:** Architectural extrapolation and hardware scalability analysis for 3D cardiac tissue simulation.

Architectural metric	Current implementation (200×200×3)	Hypothetical direct spatial mapping (Single FPGA, $200^{3}$ )	Hypothetical time-multiplexed (Single FPGA, $200^{3}$ )	Future viable architecture (Multi-FPGA + HBM, $200^{3}$ )
Total grid nodes	120, 000	8, 000, 000	8, 000, 000	8, 000, 000
Look-up tables (LUTs)	214, 652	≈14,300,000 ^a^	214, 652^b^	Distributed ( <60% per chip)
DSP slices	1, 200	≈80,000 ^a^	1, 200	Distributed
BRAM blocks (36 Kb)	480	≈32,000 ^a^	≈32,000 ^a^	External high bandwidth memory
Maximum frequency $\left(F_{\max}\right)$	125 MHz	Unroutable (timing failure)	125 MHz	≈100−150 MHz
Lattice update time	0.96 m	N/A	≈64.0 ms^c^	≈5.0−10.0 ms
Biological real-time margin	56.4× (accelerated)	N/A	0.84× (slower than biological)	≈5×

^a^ Physically impossible: Exceeds the Virtex-7, maximum capacities (2, 073, 600 LUTs, 6, 600 DSPs, 1, 470 BRAMs).

^b^ Assumes reusing the existing Processing Element (PE) array to calculate different tissue layers sequentially.

^c^ Scaling the 0.96 m update time by the 66.6 volumetric expansion factor, destroying the real-time capability required for arrhythmia detection.

In conclusion, while biologically functioning as a thin tissue slab, the 200×200×3 dimension represents an optimal hardware “sweet-spot” for a Proof-of-Concept (PoC). It provides the topological minimum to validate volumetric 3D wave dynamics, spatial routing, and transmural heterogeneities while strictly preserving the computational margins necessary for real-time critical care applications. Expanding to a $200^{3}$ whole-heart model requires a paradigm shift toward Multi-FPGA clusters interconnected via high-speed serial transceivers and High Bandwidth Memory (HBM) modules, which remains a targeted avenue for future work.

While scaling to a full $200^{3}$ geometry is clearly prohibitive for a single FPGA, one might consider intermediate depths, such as Z=10 or Z=20 , to enrich the biological dynamics. Increasing the depth to these dimensions would undeniably allow for the manifestation of more complex 3D phenomena, such as scroll wave fragmentation and richer transmural repolarization gradients. However, as demonstrated in Table 12, even these intermediate scaling factors rapidly collide with the strict hardware boundaries of the target silicon.

**TABLE 12:** Hardware feasibility limits and temporal dynamics for intermediate 3D depths on a single Virtex-7 FPGA.

Grid Dimensions	Total Nodes	Architecture Map	Est. BRAM (36 Kb)	Est. DSP Slices	Est. LUTs	Lattice Update Time	Biological Margin	Hardware Verdict
200×200×3	120, 000	Direct mapping (baseline)	480	1,200	214,652	0.96 ms	56.4×	Feasible (high margin)
200×200×10	400, 000	Direct mapping	≈1,600 ^a^	≈4,000	≈715,000	N/A (fails)	N/A	**Fails** (memory wall)
200×200×10	400, 000	Time-multiplexing (TM)	≈1,600 ^a^	1, 200^b^	214, 652^b^	≈3.20 ms ^c^	≈16.9× ^c^	**Fails** (irreducible memory wall)
200×200×20	800, 000	Direct mapping	≈3,200 ^a^	≈8,000 ^a^	≈1,430,000	N/A (fails)	N/A	Fails (BRAM and DSP exhaustion)
200×200×20	800, 000	Time-multiplexing (TM)	≈3,200 ^a^	1, 200^b^	214, 652^b^	≈6.40 ms ^c^	≈8.5× ^c^	**Fails** (irreducible memory wall)

^a^ Exceeds the maximum physical capacity of the Xilinx Virtex-7 XC7V2000T (Max BRAM: 1, 470; Max DSP: 6, 600).

^b^ TM, reuses existing arithmetic units (DSP/LUTs) maintaining them equivalent to the baseline, but biological state storage (BRAM) cannot be reduced.

^c^ Theoretical internal latency assuming perfect memory bandwidth. Off-chip memory resolution would cause massive I/O bottlenecks, nullifying these margins.

Extrapolating the hardware utilization from the baseline Z=3 model (comprising 120, 000 nodes) to Z=10 (400, 000 nodes) entails a volumetric scaling factor of ≈3.33× . While the logic and arithmetic operations might still fit within the Virtex-7 architecture under a direct spatial mapping (requiring approximately 4, 000 DSP slices, which is below the 6, 600 limit), this depth immediately triggers a critical *Memory Wall*. Specifically, the expanded state variables require roughly 1, 600 BRAM blocks, directly exceeding the 1, 470 BRAM capacity of the XC7V2000T chip.

Further increasing the depth to Z=20 (800, 000 nodes, a ≈6.66× volumetric scaling) results in a catastrophic failure in both memory and arithmetic mapping. This configuration demands an estimated 3, 200 BRAMs and 8, 000 DSP slices, overwhelming the fundamental computational resources of any single state-of-the-art FPGA in this family.

Even if Time-Multiplexing (TM) were applied to reuse the logic and DSP elements for these intermediate depths—keeping the DSP and LUT counts identical to the baseline (1, 200 and 214, 652, respectively) while proportionately increasing the lattice update time—the hardware still fails. The fundamental requirement to concurrently store the biological state variables (e.g., transmembrane potentials and recovery variables) dictates that the BRAM requirements remain largely unchanged ( ≈1,600 for Z=10 and ≈3,200 for Z=20 ). Attempting to mitigate this irreducible memory deficit by routing data to off-chip memory (e.g., DDR3/DDR4) would introduce severe I/O latency bottlenecks, destroying the simulated internal latency (e.g., 3.20 m or 6.40 m) and compromising the real-time margins. Consequently, Z=3 proves to be not just an arbitrary choice, but the optimal “topological minimum” that mathematically preserves 3D spatial coupling while remaining strictly bounded within the monolithic physical resources of a single FPGA.

While our FPGA framework successfully accelerates the electrophysiological computations, it is important to acknowledge the biophysical limitations of the proof-of-concept simulations presented in this study. The 200×200 grid utilizes a coarse spatial resolution, which introduces known numerical artifacts. As a result, the simulations exhibit non-physiological behaviors, most notably the square-like wave propagation observed in Figures 4, 7, as well as the artificial stability of multiple spiral waves within a restricted domain. We emphasize that these phenomena are computational artifacts rather than accurate representations of clinical cardiac biophysics. The primary purpose of this specific simulation was to serve as a computational benchmark to stress-test the hardware architecture, real-time processing pipeline, and memory management under the constraints of the current FPGA platform. However, the proposed hardware accelerator method is inherently scalable. Future implementations utilizing next-generation FPGAs with expanded memory and logic resources will readily accommodate the finer spatial resolutions required to eliminate these artifacts, thereby providing high-fidelity, physiologically accurate simulations for the cardiac community.

It is worth noting that the computational acceleration achieved in this study is partly attributed to the mathematical simplicity of the Aliev-Panfilov (AP) model. As a phenomenological model, the AP model simplifies the complex microscopic ionic mechanisms into two governing state variables, which drastically reduces the localized hardware resource footprint (DSPs and BRAMs) per computational node compared to biophysically detailed models such as the ten Tusscher or O’Hara-Rudy models. For instance, implementing a full biophysical model involves solving over a dozen state variables and highly non-linear gating equations containing transcendental functions. On an FPGA platform, a direct mapping of such detailed models would lead to severe resource inflation, limiting the maximum achievable parallel lattice size on a single chip. Therefore, the AP model served as an essential baseline to validate the scalability of our proposed 3D parallel routing architecture and multi-precision framework. Crucially, the underlying spatial discretization, parallelization scheme, and memory-streaming architecture developed in this work are inherently model-agnostic. Future iterations will focus on incorporating these complex ionic models by employing hardware-efficient optimization techniques, such as Coordinate Rotation Digital Computer (CORDIC) and Piecewise Linear Approximation (PWL), to balance physiological fidelity with large-scale spatial simulation.

## 8 Comparison with state-of-the-art and discussion

To adequately contextualize our findings and address the current landscape of FPGA-based cardiac modeling, Table 13 presents a comprehensive comparative analysis. To the best of our knowledge, this work represents the first exact FPGA implementation of the 3D Aliev-Panfilov (AP) model. Consequently, we benchmark our proposed 3D cardiac digital twin-inspired frame work against the most closely related state-of-the-art works, including single-cell and 1D network models (Ghanbarpour et al., 2025; Ghanbarpour et al., 2024; Mansouri et al., 2022; Naderi et al., 2026; Liu et al., 2024), as well as advanced FPGA-based cardiac arrhythmia classification systems (Ghanbarpour et al., 2026; Chandrasekaran et al., 2025). While direct one-to-one comparisons are challenging due to differing dimensionalities and objectives, evaluating these works collectively reveals several critical insights regarding the validity, applicability, advantages, and inherent hardware trade-offs of the proposed system.

- Massive Scalability and Structural Advantages: The most prominent advantage of our proposed architecture is its spatiotemporal dimensionality. Existing FPGA implementations are largely confined to single isolated cells (using as few as 102 LUTs (Mansouri et al., 2022)) or 1D propagation cables limited to approximately 4,500 nodes (Ghanbarpour et al., 2025). In stark contrast, our system models a massive 3D lattice comprising 120,000 fully coupled nodes. This leap to 3D dynamics is not merely a quantitative expansion but a qualitative necessity, as it enables the simulation of complex, organ-level arrhythmic phenomena—such as 3D spiral wave breakup—which are mathematically impossible to observe in 1D or single-cell environments.
- Physiological Validity vs. Data-Driven Models: When compared to deep-learning-based cardiac classifiers (Chandrasekaran et al., 2025) that achieve high accuracy (e.g., 99% ) relying entirely on clinical datasets like MIT-BIH, our system offers a different paradigm of physiological validity. Rather than acting as a black-box anomaly detector, the proposed digital twin-inspired frame work provides a biophysically grounded, mechanistic simulation of action potential propagation. Furthermore, by adopting an optimized 18-bit fixed-point precision strategy, we managed to maintain a highly reliable physiological correlation of 98.5% with floating-point software baselines, effectively balancing precision with the stringent resource constraints of the FPGA.
- Hardware and Power Trade-offs: The substantial increase in scalability naturally incurs predictable hardware costs. The proposed 3D lattice consumes 214,652 LUTs (10.35%) , 157,840 FFs (3.81%) , and 1,200 DSPs (18.18%) on the Virtex-7 XC7V2000T platform. Similarly, the total power consumption is 5.42 W, which is notably higher than the ∼0.3 W reported for smaller 1D networks (Ghanbarpour et al., 2025; Ghanbarpour et al., 2024). However, this is a highly justified trade-off, reflecting the immense parallel computational load required to update 120,000 nonlinear differential equations simultaneously at every time step.
- Performance and Real-Time Applicability: The dense interconnection network required for a fully coupled 3D spatial grid restricts the maximum operating frequency $\left(F_{\max}\right)$ to 125 MHz, compared to the ∼600 MHz achievable in highly localized 1D arrays (Ghanbarpour et al., 2025). Despite this routing-induced frequency limitation, the highly parallel architecture delivers a full lattice update latency of exactly 0.96 m. This sub-millisecond execution time is the most critical metric for applicability, as it strictly guarantees real-time operation. Consequently, the proposed system is exceptionally well-suited for Hardware-In-the-Loop (HIL) environments, enabling real-time closed-loop testing of pacemakers and continuous predictive clinical modeling.

**TABLE 13:** Comprehensive Comparative Analysis of the Proposed 3D Cardiac digital twin-inspired frame work with State-of-the-Art FPGA-Based Biological and Biosignal Implementations.

Metric/Feature	SA Node (CORDIC) (Ghanbarpour et al., 2024)	Purkinje cell (Base-2) (Mansouri et al., 2022)	Purkinje net (CORDIC) (Ghanbarpour et al., 2025)	SA Node cell (Low Cost) (Naderi et al., 2026)	UKF filter (Denoising) (Ghanbarpour et al., 2026)	Purkinje Cell (Base-2) (Liu et al., 2024)	Arrhythmia detection (classifier) (Chandrasekaran et al., 2025)	Proposed work
Application domain	Cardiac pace	Single cell	Purkinje net.	Pacemaker	Signal Proc	Purkinje cell	Arrhythmia Det.	3D cardiac twin
Dynamics type	1D network	Single cell	1D network	1D network	Filtering	Single cell	Classification	3D spatiotemporal
Numerical model	YNI model	Noble model	Noble model	YNI model	UKF algorithm	Noble model	Deep learning	Aliev-Panfilov
Scalability (nodes)	100 cells	1 cell	4,500 cells	103 cell	5 channels	1 cell	N/A	120,000 nodes
Hardware platform	Zynq XC7Z010	Spartan-3 XC3S50	Virtex-7	Zynq XC7Z010	Zynq-7000	Spartan-3 XC3S50	Zynq Ultra+	Virtex-7
Precision strategy	41-bit Fixed-Pt	51-bit Fixed-Pt	36-bit Fixed-Pt	51-bit Fixed-Pt	Fixed-point	60-bit Fixed-Pt	Fixed-point	18-bit fixed-Pt
Max Freq. (MHz)	472.769	162.273	598.724	241.557	194.10	242.361	N/A	125
LUT utilization	17,600	102	21657	1704	762	81	53,810	214,652
FF utilization	35200	168	25908	1491	536	143	24,026	157,840
DSP blocks	0	0	0	0	3	0	131	1,200
Power cons. (W)	0.291	N/A	0.319	0.285	0.016	0.284	1.02	5.42
Latency/Step	19.3 ns	N/A	N/A	N/A	4.3 ns	N/A	3.83 m	6
Validation method	RMSE: 1.014	RMSE: 2.99	RMSE: 0.103	RMSE: 0.43	Corr: 98.89%	Corr: 96.46%	Acc: 99.01%	Corr: 98.54%–99.12%
Physiological validity	Single action potential	Single action potential	1D propagation	Single action potential	N/A	Single action potential	Clinical ECG (MIT-BIH)	3D spiral breakup

In summary, while the transition from 1D to massive 3D modeling demands higher resource utilization and power, the proposed system successfully bridges the gap between isolated cellular models and organ-level biological emulations, offering unprecedented real-time insights into 3D cardiac electrophysiology.

## 9 Conclusion

In this study, we successfully developed and validated a high-performance 3D cardiac digital twin-inspired frame work. The hardware was validated through (1) numerical cross-verification against a MATLAB reference (98.5% correlation) and (2) physiological grounding using the Aliev-Panfilov model to replicate clinical spiral breakup patterns. By implementing the Aliev-Panfilov model on a Xilinx Virtex-7 FPGA, we demonstrated that a multi-precision fixed-point approach can achieve a remarkable balance between computational throughput and physiological accuracy. The proposed system’s ability to process 120,000 nodes in under 1 m—providing a 56-fold speedup over biological time—establishes a robust foundation for “digital twin-inspired frame work” technology in cardiology. Unlike software-based solutions, our hardware-centric approach enables the instantaneous detection of spiral wave turbulence and the autonomous classification of arrhythmic states (PS, QS, and ST). This sub-millisecond responsiveness is critical for closed-loop interventions, where the latency of traditional processors often fails to suppress chaotic fibrillation effectively. This research proves that FPGA-based hardware accelerators can provide the necessary deterministic performance for complex biological modeling. Future work will focus on integrating patient-specific anatomical data and expanding the control algorithms to handle multi-focal arrhythmias, further bridging the gap between theoretical electrophysiology and real-time clinical applications. Ultimately, this work serves as a critical step towards the realization of hardware-accelerated digital twins for cardiac care, with profound implications for the future development of intelligent biosensing and real-time monitoring systems for arrhythmia management.

## Data Availability

The raw data supporting the conclusions of this article will be made available by the authors, without undue reservation.
