# Supplementary figures and images for: Real-time 3D cardiac tissue implementation for arrhythmia detection and management
Source: Front Bioeng Biotechnol. 2026 Jul 3;14:1840143. doi: 10.3389/fbioe.2026.1840143 (PMC13375795; doi:10.3389/fbioe.2026.1840143)

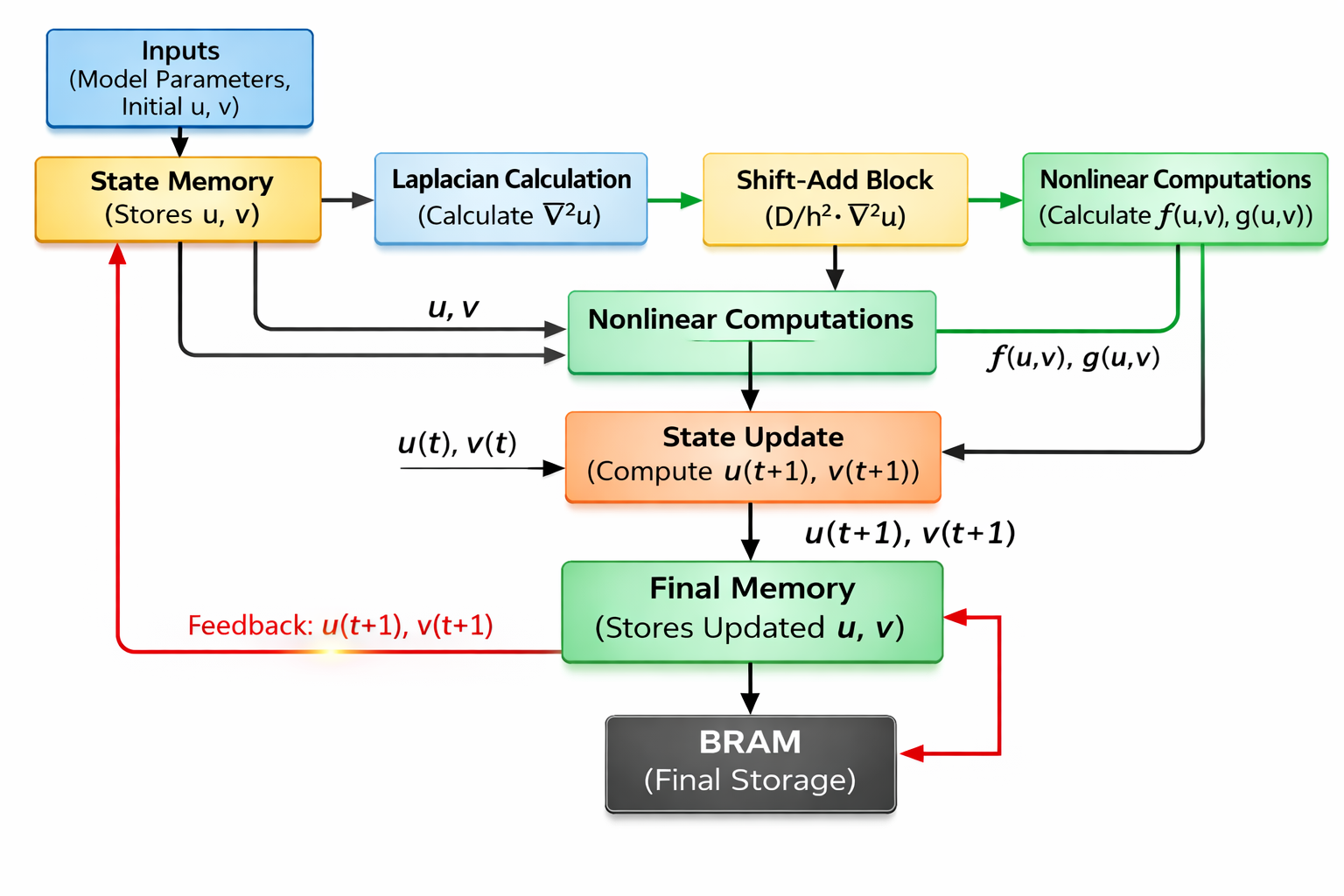

Supplement: Supplementary file 1 [file DataSheet1.zip › Highlighted/architecture_single.png]

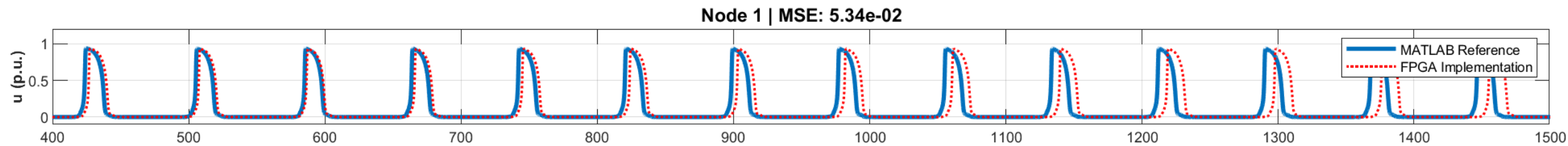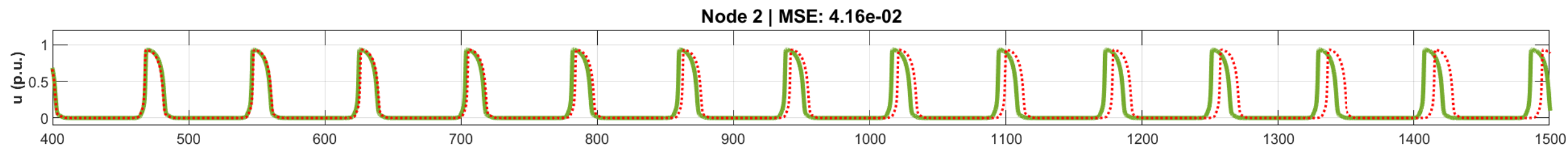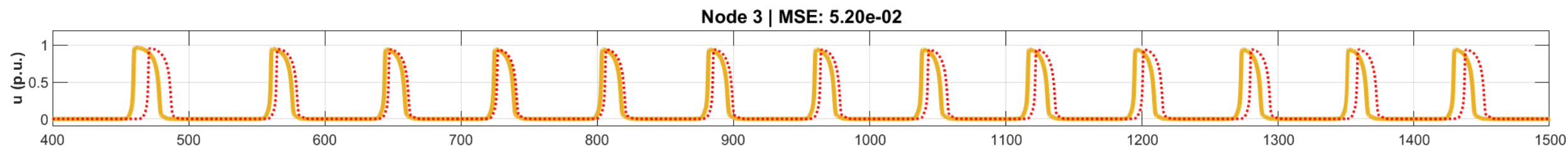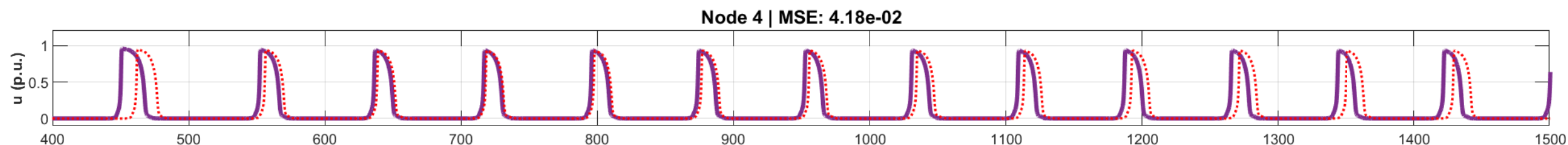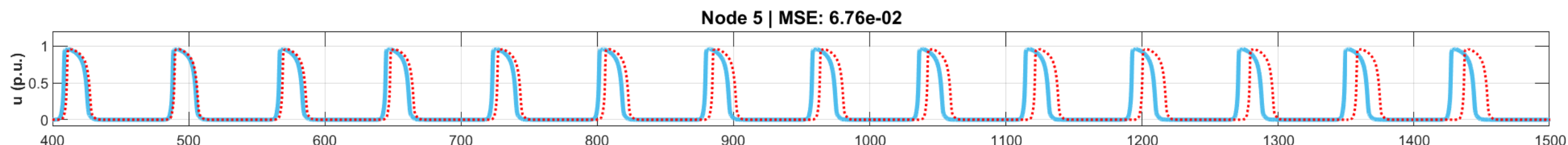

Time (ms)

Supplement: Supplementary file 1 [file DataSheet1.zip › Highlighted/fpga_simulation_PS-eps-converted-to.pdf]

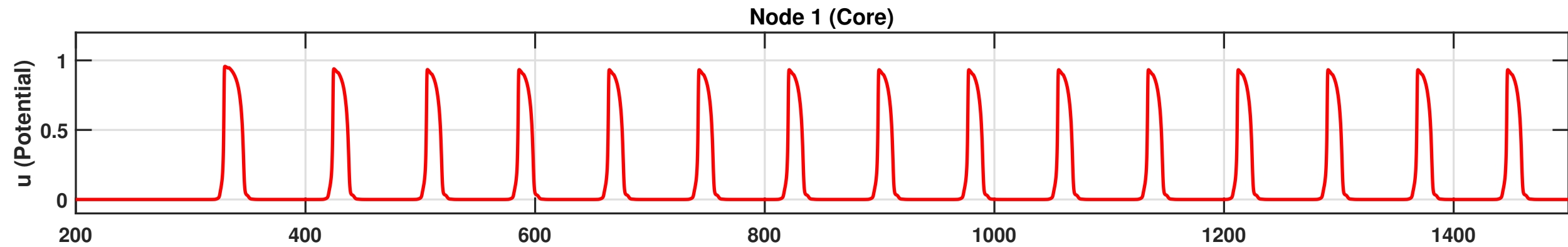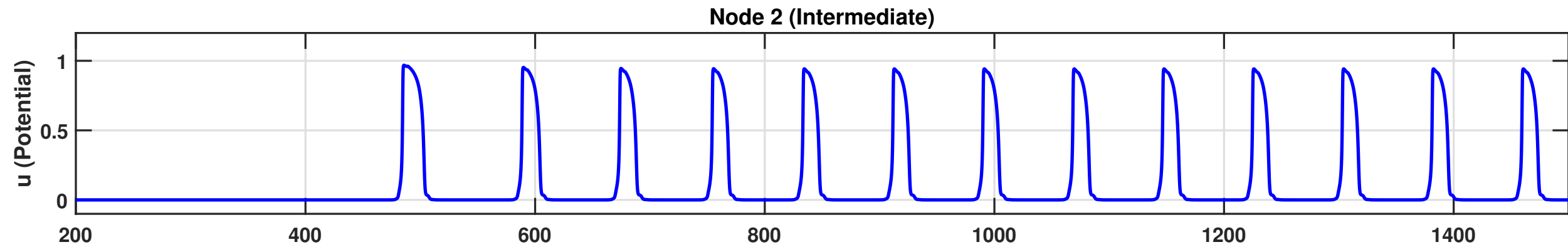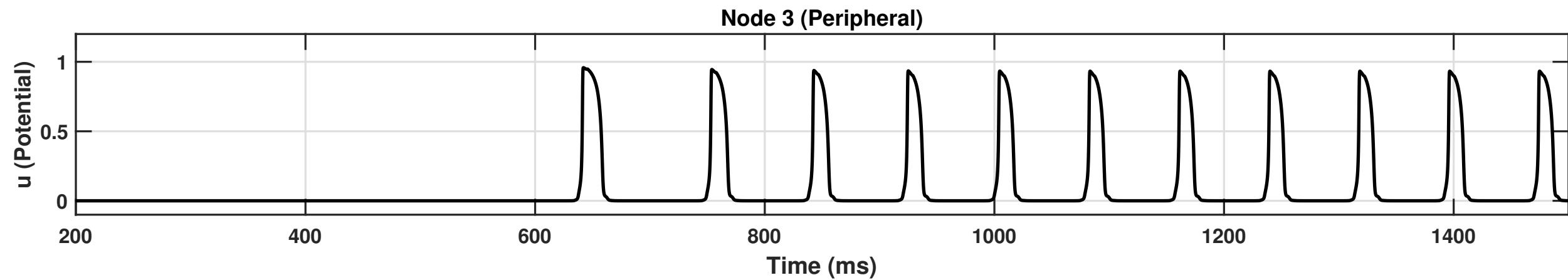

Supplement: Supplementary file 1 [file DataSheet1.zip › Highlighted/PS-eps-converted-to.pdf]

**Layer Z = 1**

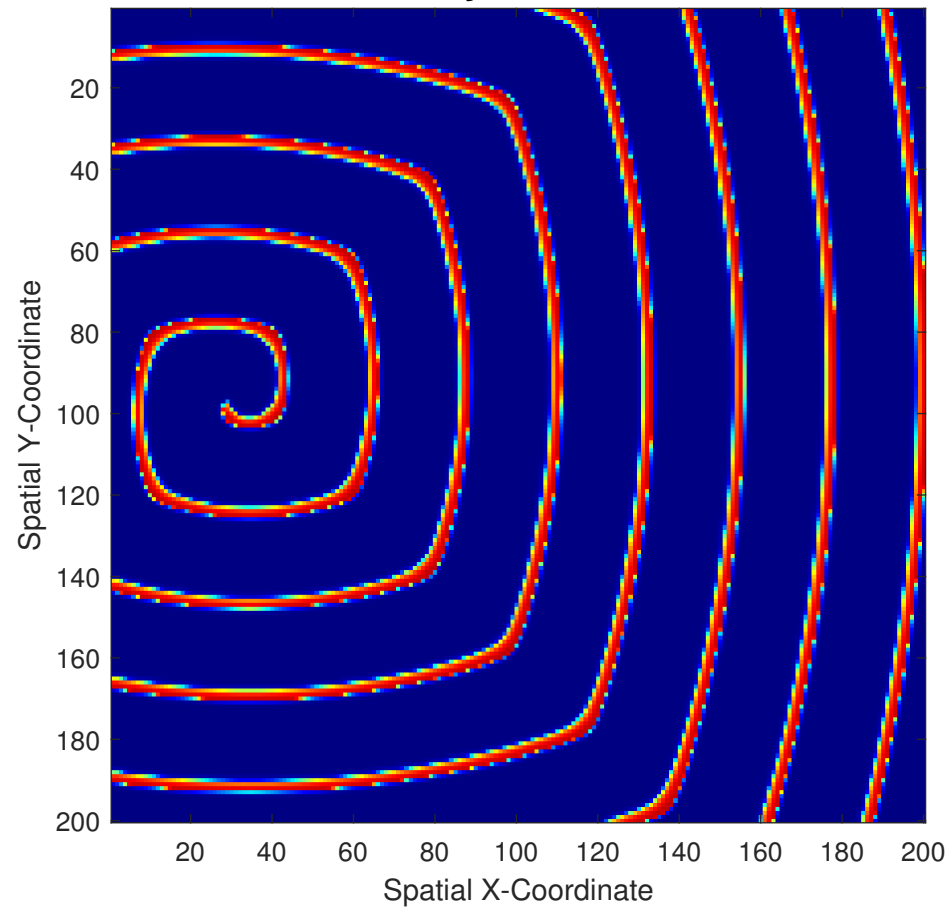

**Layer Z = 2**

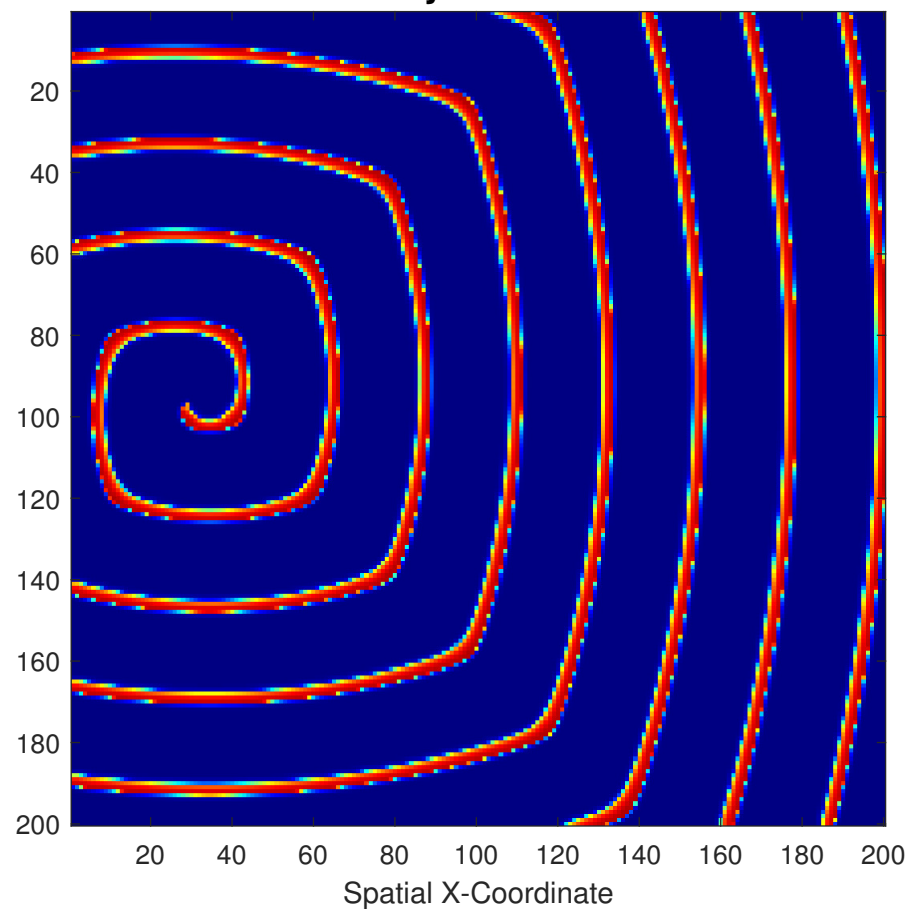

**Layer Z = 3**

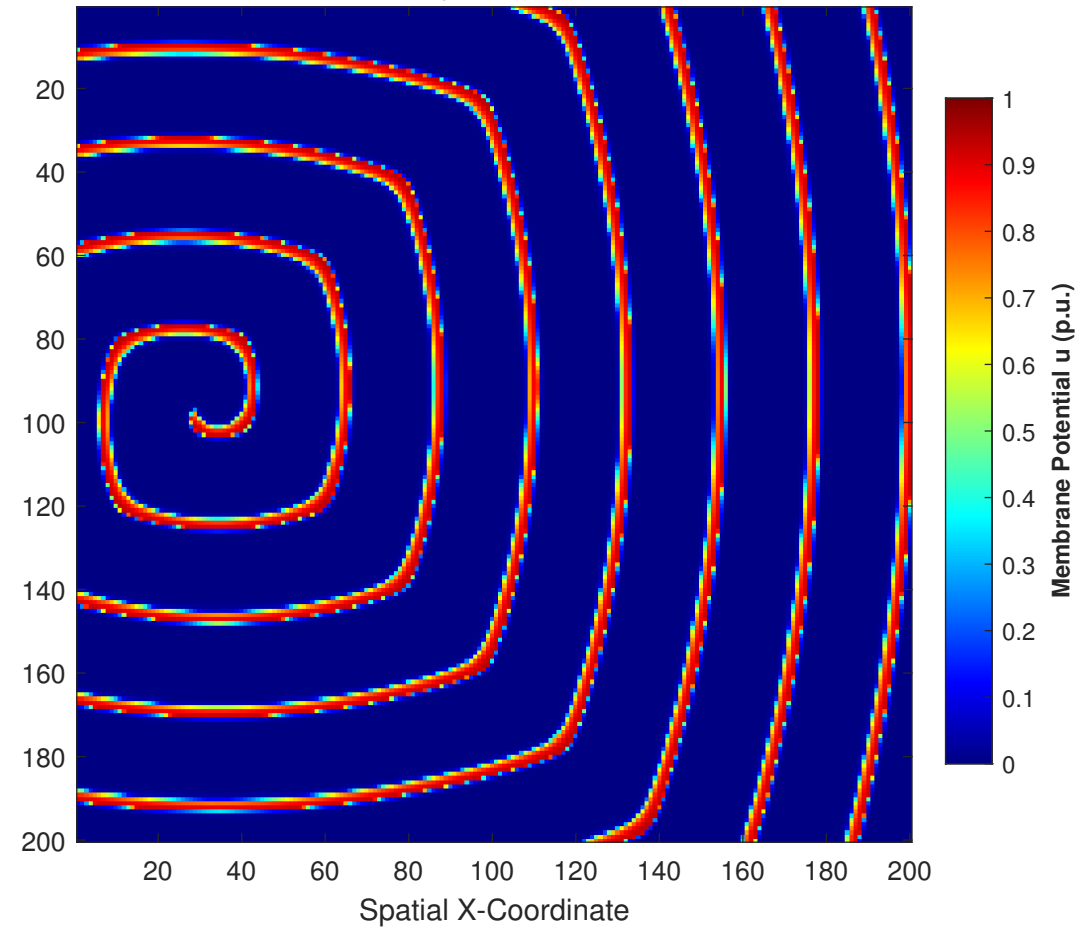

Supplement: Supplementary file 1 [file DataSheet1.zip › Highlighted/Spatiotemporal-eps-converted-to.pdf]

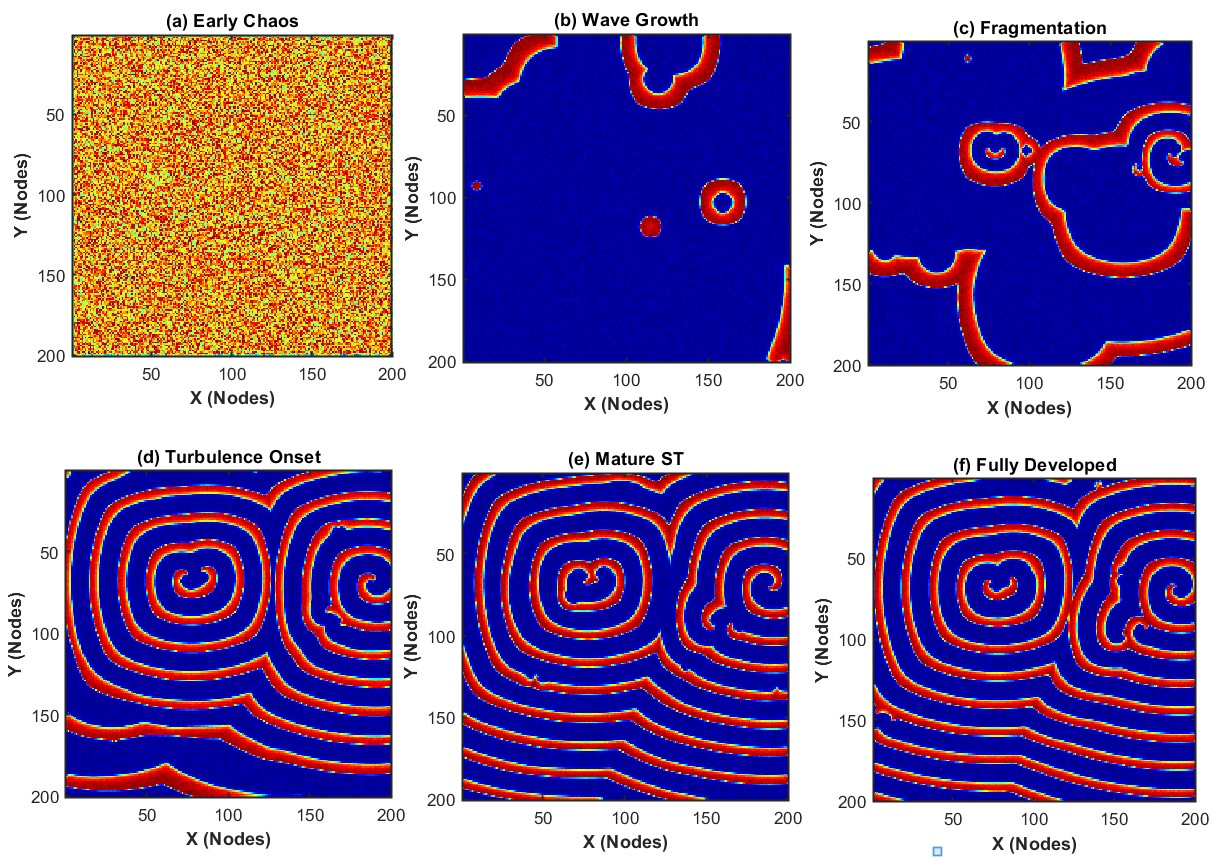

Supplement: Supplementary file 1 [file DataSheet1.zip › Highlighted/ST.jpg]

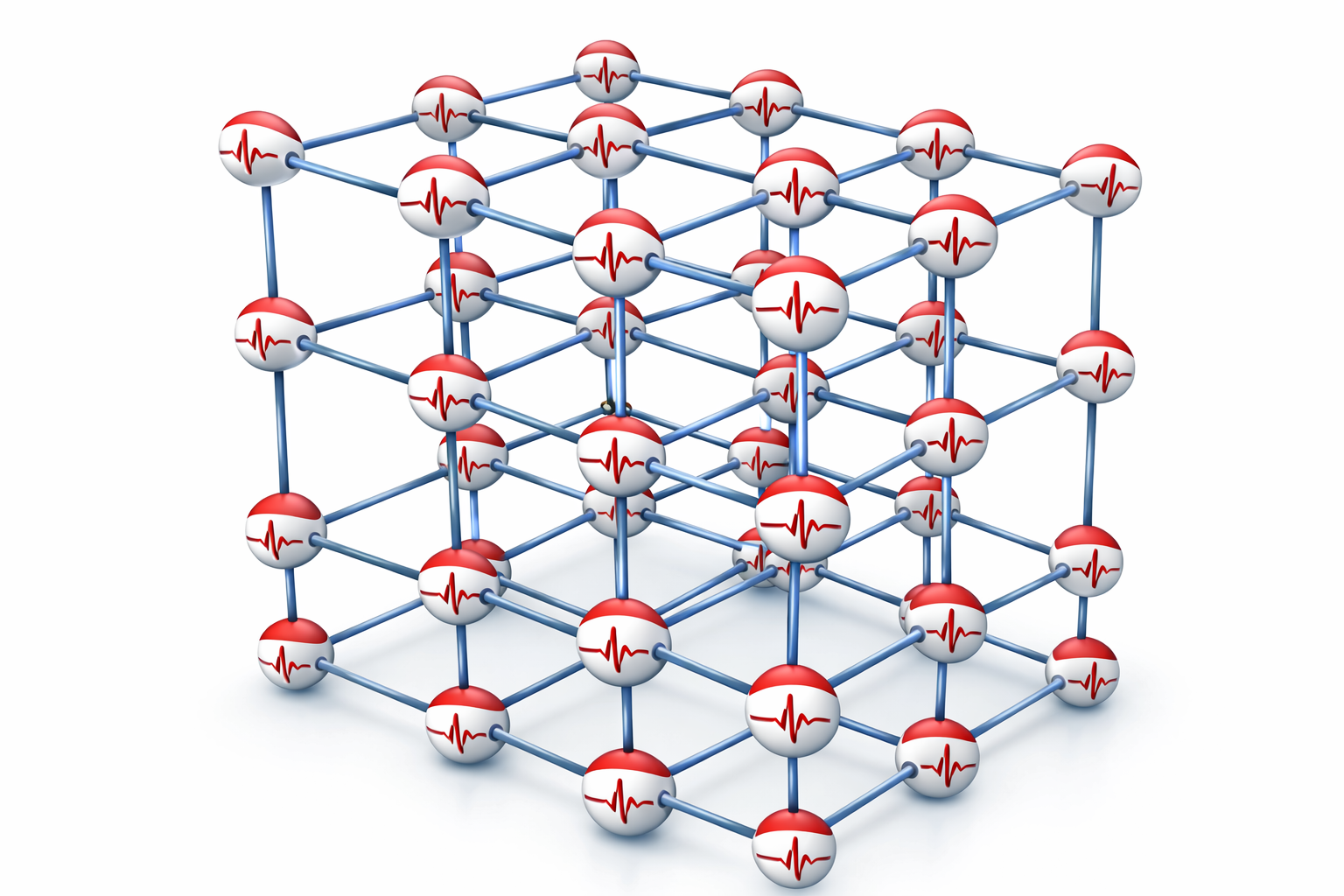

Supplement: Supplementary file 1 [file DataSheet1.zip › Highlighted/topology.png]

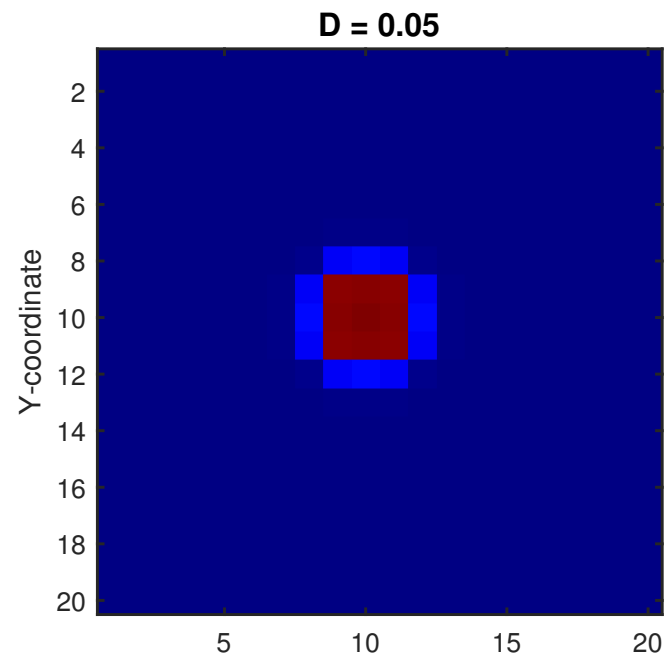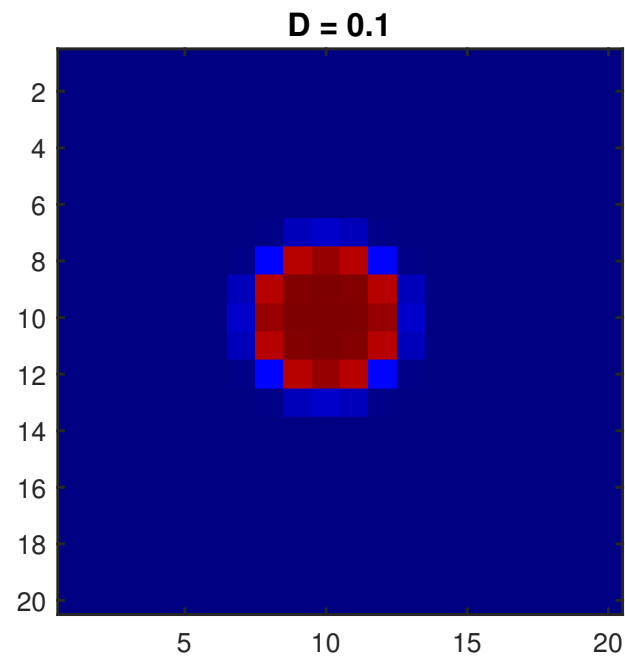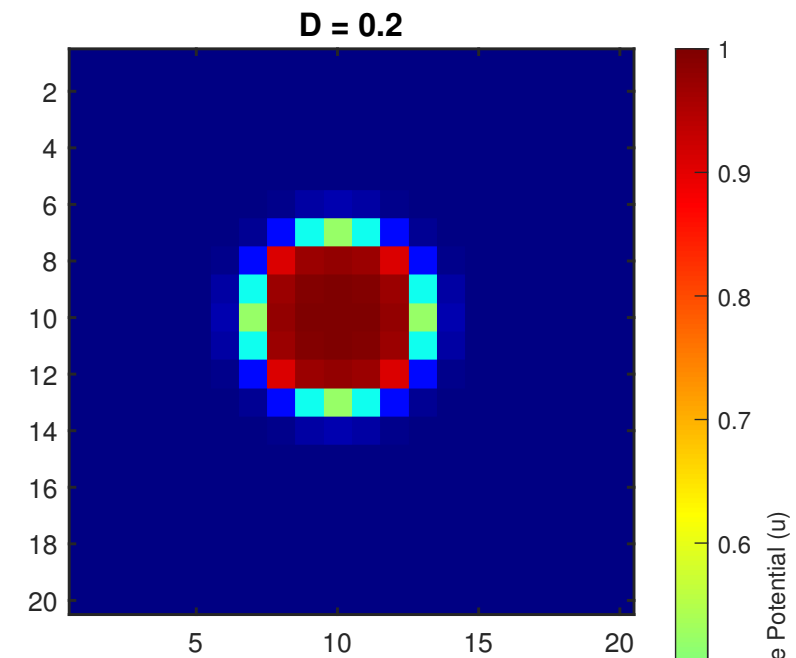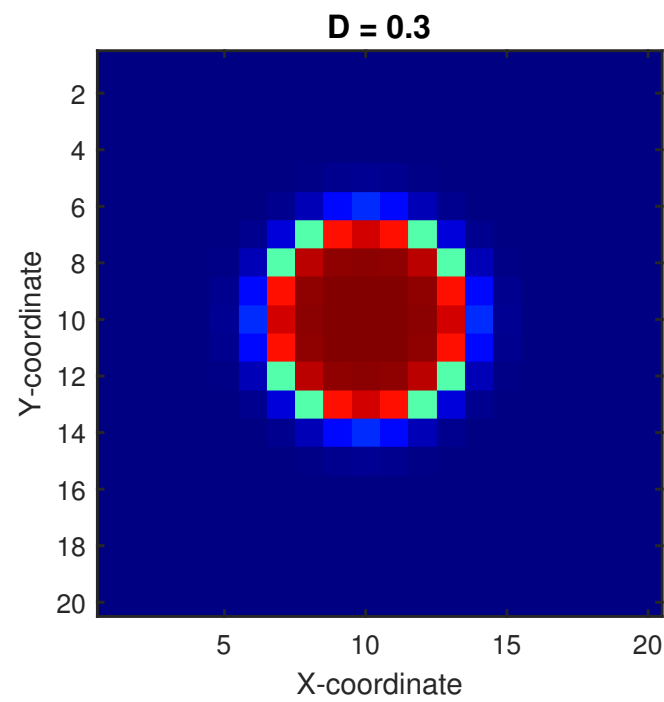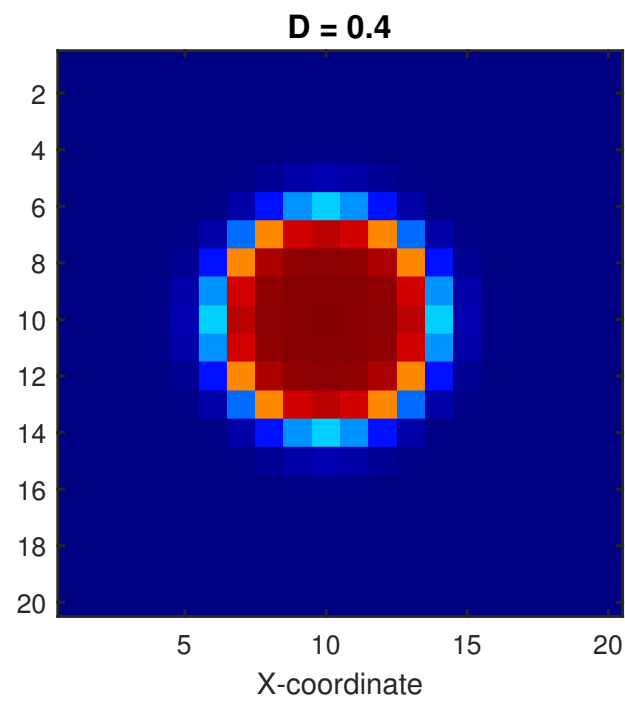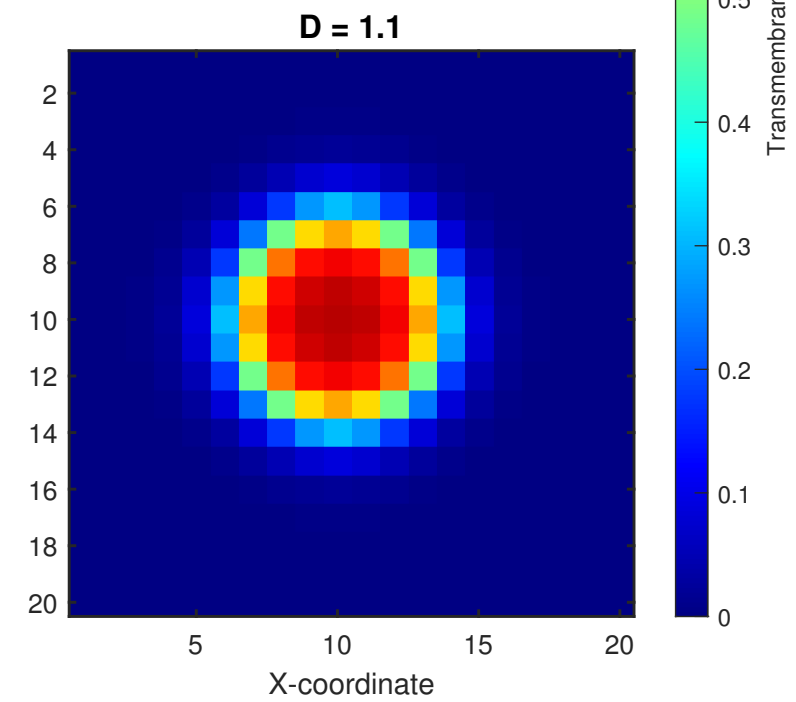

Supplement: Supplementary file 1 [file DataSheet1.zip › Highlighted/wave-eps-converted-to.pdf]
